# Development and characterization of a recombinant silk network for 3D culture of immortalized and fresh tumor‐derived breast cancer cells

**DOI:** 10.1002/btm2.10537

**Published:** 2023-05-11

**Authors:** Caterina Collodet, Kelly Blust, Savvini Gkouma, Emmy Ståhl, Xinsong Chen, Johan Hartman, My Hedhammar

**Affiliations:** ^1^ Division of Protein Technology School of Biotechnology, KTH Royal Institute of Technology Stockholm Sweden; ^2^ Department of Oncology‐Pathology Karolinska Institutet Stockholm Sweden; ^3^ Department of Clinical Pathology and Cancer Diagnostics Karolinska University Hospital Stockholm Sweden

**Keywords:** 3D model, breast cancer, FN‐silk network, MCF‐7, MDA‐MB‐231, RNA‐seq, SK‐BR‐3

## Abstract

Traditional cancer models rely on 2D cell cultures or 3D spheroids, which fail to recapitulate cell‐extracellular matrix (ECM) interactions, a key element of tumor development. Existing hydrogel‐based 3D alternatives lack mechanical support for cell growth and often suffer from low reproducibility. Here we report a novel strategy to make 3D models of breast cancer using a tissue‐like, well‐defined network environment based on recombinant spider silk, functionalized with a cell adhesion motif from fibronectin (FN‐silk). With this approach, the canonical cancer cells SK‐BR‐3, MCF‐7, and MDA‐MB‐231, maintain their characteristic expression of markers (i.e., ERα, HER2, and PGR) while developing distinct morphology. Transcriptomic analyses demonstrate how culture in the FN‐silk networks modulates the biological processes of cell adhesion and migration while affecting physiological events involved in malignancy, such as inflammation, remodeling of the ECM, and resistance to anticancer drugs. Finally, we show that integration in FN‐silk networks promotes the viability of cells obtained from the superficial scraping of patients' breast tumors.

## 1 INTRODUCTION

Breast cancer is the most common cancer among women worldwide, excluding nonmelanoma skin cancer.^1^ Breast cancer is a heterogeneous disease composed of several subtypes, each with different morphological and clinical implications.^2^ To tailor therapies, patients are routinely classified by assessing the expression of three histological markers (i.e., ERα, HER2, and PR). In the past decades, several studies identified collections of genes that allow further patient stratification.^3^

Despite significant advances in the field, there is still a need to find new drugs. With many candidates failing in clinical trial phases,^4^ developing more reliable preclinical models is crucial. Traditionally, the first stages of preclinical research rely on cells grown on a flat surface, lacking the structure of tumors and tissues.^5^ Likely due to these oversimplified conditions, in vitro models often fail to recapitulate the in vivo response.^6^, ^7^ Three‐dimensional (3D) models have been suggested as a bridge between in vitro and in vivo models since they better mimic the complexity of the tumor microenvironment.^8^, ^9^

Three‐dimensional models can be divided into scaffold‐free models, where cells are forced to self‐aggregate forming tumor spheroids, and scaffold‐based systems, with cells growing onto extracellular matrix (ECM)‐mimetic biomaterials.^10^ Scaffold‐free alternatives have been suggested to recapitulate the oxygen, nutrients, and pH gradients of solid human tumors^11^ but lack the cell‐ECM interaction component. The alternative scaffold‐based systems often rely on materials with ill‐defined composition, having batch‐to‐batch variability and limited reproducibility.^12^ Additionally, it is often difficult to obtain good seeding efficiency due to cells' inability to spread homogeneously through rigid structures.^13^ These drawbacks have driven the search for alternatives, one of which is the recombinantly produced spider silk protein, based on the four poly‐Ala/Gly‐rich regions and the nonrepetitive C‐terminal domain of a spidroin, 4RepCT, genetically functionalized to harbor the Arg‐Gly‐Asp (RGD) containing cell binding motif from fibronectin (FN) for optimal cell adhesion,^14^, ^15^, ^16^ herein referred to as FN‐silk. Cells can be added to an FN‐silk solution before its self‐assembly, which is then carried out under physiological conditions and leads to the formation of a network mimicking the fibrous part of the ECM with homogeneously integrated mammalian cells.^17^ Furthermore, FN‐silk can be easily sterilized, is well tolerated in vivo, and is of non‐animal origin.^18^

In this study, we developed a new technique to generate floating networks of FN‐silk containing breast cancer cells. This system extends our previous work in which various cell types, including endothelial cells, fibroblasts, keratinocytes, and pluripotent stem cells,^19^ were successfully cultured on FN‐silk. For the current study, cell lines representative of the three main subtypes of breast cancer HER2‐overexpression, luminal‐like, and triple‐negative, respectively SK‐BR‐3, MCF‐7, and MDA‐MB‐231 were used to compare culture in FN‐silk to traditional 2D culture. We assessed the proliferation rate and expression of the markers routinely used to classify breast tumors and investigated by RNA‐sequencing (RNA‐seq) how FN‐silk modulates the transcriptional landscape of MCF‐7 and MDA‐MB‐231. The bioinformatics analysis revealed that culture in FN‐silk network affected the expression of genes related to pathophysiological cancer processes such as cell adhesion, migration, and inflammatory signaling. We also demonstrated that FN‐silk networks are highly adaptable, allowing the growth of novel breast cancer cells, such as the clinically relevant PB and Wood, as well as cells obtained from fresh tumors.

## 2 MATERIALS AND METHODS

Full details are available in the Supplementary Information and list of RT‐qPCR primers can be found in Table 1.

**TABLE 1:** RT‐qPCR primers. List of the human primers used in this study completed of gene abbreviation, gene name, and forward/reverse sequences.

Gene abbreviation	Gene name	Forward primer	Reverse primer
*AP1G2*	Adaptor related protein complex 1 gamma 2 subunit	CTCATGAAGCTCAGCACTCG	AGCCCTCATGTGGTCGTATT
*B2M*	Beta‐2 microglobulin	TGCTCGCGCTACTCTCTCTTT	TCTGCTGGATGACGTGAGTAAAC
*CD44*	CD44 molecule	CGGACACCATGGACAAGTTT	TGGAATACACCTGCAAAGCG
*CDH2*	N‐cadherin	TGCACAGATGTGGACAGGAT	CCACAAACATCAGCACAAGG
*CDH24*	Cadherin 24	GTAGGCCAGATCTCCGCG	GTGCTGCTGTATGGATGGTG
*CLDN3*	Claudin 3	AGGCGTGCTGTTCCTTCTC	CACCACGGGGTTGTAGAAGT
*CNKSR1*	Connector enhancer of kinase suppressor of Ras 1	CAACATCCTGGTCTGCTGC	TGGTGGTGTGAATTTCTAGGC
*DSC2*	Desmocollin 2	TGATTTAGCCCAGCAGAACC	CCTCCGTTTTTGATTCCTGA
*ERBB2*	Human epidermal growth factor receptor 2	TGGCTCAGTGACCTGTTTTG	GGCATGTAGGAGAGGTCAGG
*ESR1*	Estrogen receptor alpha	TCTGGCGCTTGTGTTTCAAC	GCTACGAAGTGGGAATGATGAAAG
*GAPDH*	Glyceraldehyde‐3‐phosphate dehydrogenase	ACAGTCAGCCGCATCTTCTT	CATGGTGTCTGAGCGATGTG
*ITGAV*	Integrin subunit alpha V	TTGCCCTCAGTGAAGGAGAT	AGCACTGAGCAACTCCACAA
*JAM2*	Junctional adhesion molecule 2	GCCAAAACCTGGAAGAGGAT	CCACAGTTCCACTCAGAGCA
*MIR503HG*	MIR503 host gene	TAAGGGGAGAGGAAGGGTGA	CGGGCTTGGTCTTTCAGGAA
*MKI67*	Marker of proliferation Ki‐67	AGCCCCAACCAAAAGAAAGT	TTTGTGCCTTCACTTCCACA
*MMP14*	Matrix metallopeptidase 14	GTGACGGGAACTTTGACACC	TTATTCCTCACCCGCCAGAA
*NANOG*	Nanog homeobox	TTTGTGGGCCTGAAGAAAAC	CAGATCCATGGAGGAAGGAA
*OCT4*	Octamer‐binding transcription factor 4	AGTTTGTGCCAGGGTTTTTG	TTGTGTTCCCAATTCCTTCC
*PGR*	Progesterone receptor	TCCTTACCTGTGGGAGCTGT	CGATGCAGTCATTTCTTCCA
*SERPINA1*	Serpin family A member 1	AGGAGCTTGACAGAGACACA	TCTTCCTCGGTGTCCTTGAC
*SOX2*	Sex determining region Y‐box 2	CCGGTACGCTCAAAAAGAAA	AGTGTGGATGGGATTGGTGT
*TFRC*	Transferrin receptor	CATTTGTGAGGGATCTGAACCA	CGAGCAGAATACAGCCACTGTAA
*ZNF512B*	Zinc finger protein 512B	TGAAGCAGATGGGACGGC	CCGCTGGTGGTACTGGTAG
*ZO‐1*	Tight junction protein 1	CGTCCTTTTCCTGCTTGACC	TCTGATTCTACAATGCGACGA

### 2.1 FN‐silk network formation

The FN‐silk network was created using a silk protein functionalized with a motif from fibronectin, FN‐silk (3 mg/mL in PBS, endotoxin level below 200 EU/ml), provided by Spiber Technologies AB. For each construct, 10,000 cells from a cell concentrate of 5,900 cells/μl were gently mixed with 8.3 μl FN‐silk to form a 10 μl droplet. The droplet was placed on a polytetrafluoroethylene (PTFE) mold, previously anchored at the bottom of a 24‐well plate. A foam was formed by rapidly pipetting air into the droplet. Constructs were stabilized by incubation for 20 min at 37°C. After, the FN‐silk networks were transferred to a hydrophobic 96‐well plate (Sarstedt, 83.3924.500) using a round‐shaped stainless steel micro spoon (Sigma, Z648299). To facilitate the transfer, medium was added to the 24‐well plate and the 96‐well plate. After transferring, the medium was removed, and a 3D‐printed cap was placed on the plate. The cap was then connected to a switched‐off Vacusafe aspiration system (Integra). The Vacusafe was switched on for 1 min to allow for the creation of a pressure difference. Then the tubing part connected to the cap was quickly disconnected, allowing the pressure to release. This procedure was repeated twice. Fresh medium was added, and the constructs were kept in culture for 7 days. The medium was changed every other day. 2D controls were created by plating 10,000 cells per well on 96‐well plate TC treated (Thermo Fisher, 174929).

### 2.2 Bioinformatic analysis

The gene ontology was performed using the over‐representation analysis function for biological processes in Web‐Gestalt.^20^ Additionally, a gene set enrichment analysis (GSEA) was done by submitting as a custom database the gene list of Nanostring nCounter® Breast Cancer 360. The transcription factor (TF) prediction was performed using i‐*cis*Target^21^ and the upstream regulator function by QIAGEN Ingenuity Pathway Analysis.^22^ Differentially expressed genes (DEGs) comparing MCF‐7 and MDA‐MB‐231 grown in FN‐silk networks for a week with their counterpart kept in 2D were visualized as volcano plots using the ggplot2 package in R. The significance thresholds were set to the adjusted *p*‐value <0.05 and the log2 fold‐change values ≥| ± 0.38|. The top 10 DEGs were highlighted (dark gray), and several important genes were marked (black).

The code used to create the volcano plots is available on GitHub (https://github.com/kblust/vulcanoplot_breastcancer2Dvs3D).

## 3 RESULTS

### 3.1 The FN‐silk networks offer an ECM‐like environment for cells growth

**FIGURE 1:**
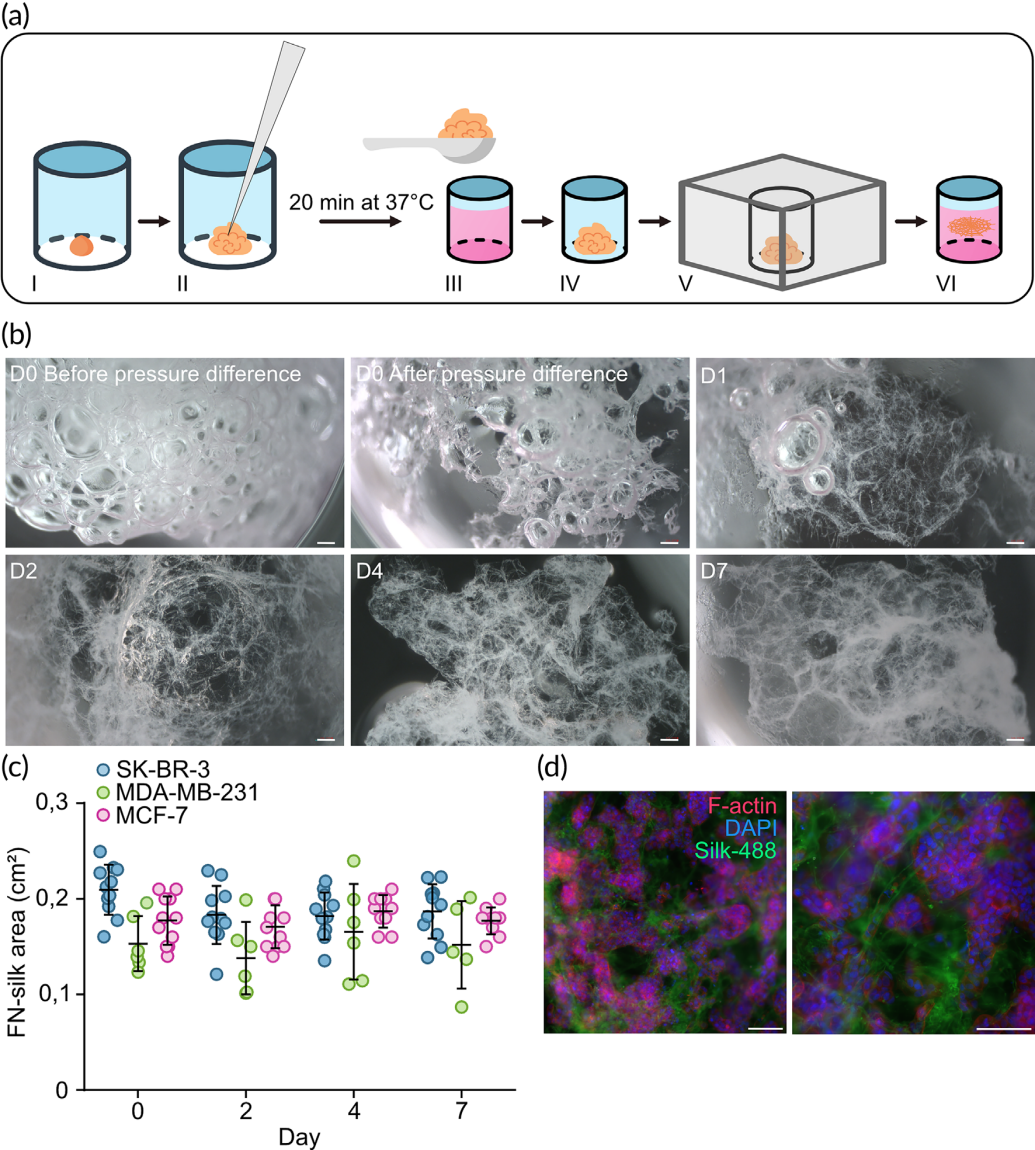
Fibronectin (FN)‐silk network cancer model formation, network morphology, and area. (a) Cartoon depicting the protocol to form FN‐silk network with cancer cells. Briefly, a solution comprising FN‐silk and a cell suspension is deposited on a PTFE‐coated well (I), self‐assembly of FN‐silk is promoted by air pipetting in the solution (II) after 20 min of stabilization at 37°C in the cell incubator, scaffolds are transferred with a micro spoon into the well of a 96‐well plate (III), the medium is removed (IV), and a pressure difference is applied (V), the medium is added, and FN‐silk networks are kept in culture for 7 days (VI). (b) Macroscopic brightfield pictures of the FN‐silk network on day 0 before and after pressure difference is applied and on days 1, 2, 4, and 7. Three independent experiments were performed, each with 8 technical replicates. Scale bar: 203 μm. (C) The area of FN‐silk network constructs generated using the cell lines MCF‐7 (pink), MDA‐MB‐231 (green), and SK‐BR‐3 (blue) is calculated by analyzing stereo microscope images taken at various time points (i.e., days 0, 2, 4, and 7). Two independent experiments were performed for each cell line, leading to 11 replicates for MDA‐MB‐231, 6 for SK‐BR‐3, and 9 for MCF‐7. Average and standard deviations are shown for each time point, and individual values are also indicated. (d) F‐Actin staining on MCF‐7 cultured for 7 days on a scaffold made using FN‐silk conjugated with 488‐fluorophore. Overlay image of F‐Actin (red), nuclei (DAPI, blue), and FN‐silk (green). Two different magnifications are shown, 10× (left) and 20× (right). *N* = 2 with three technical replicates. Scale bar: 100 μm.

Figure 1a describes the various steps required to form the FN‐silk networks with breast cancer cells. An initial drop containing 10,000 breast cancer cells and FN‐silk protein solution was placed onto a sheet of hydrophobic PTFE in a culture well (step I). Air bubbles were introduced through rapid pipetting, allowing the creation of foam with multiple liquid‐air interfaces in which FN‐silk assembled into sheets around each air bubble (step II). The construct was placed in a cell incubator at 37°C for 20 min to stabilize the structure. This quick way to initiate the network formation, which was carried out at room temperature, allowed cells to homogeneously distribute through the scaffold from its first phases. Afterward, each foam was transferred to a well of a 96‐well plate containing medium, using a micro spoon (step III). The medium was then removed (step IV), and, to promote the release of the air bubbles, thereby yielding a network, a pressure difference was applied by placing a 3D‐printed cap on top of the plate and connecting it to a standard medium aspiration tool for 2 min (step V). Finally, the medium was added, and cells were kept in culture for 7 days within the FN‐silk networks.

Brightfield pictures were taken at various time points to monitor how quickly the constructs would get to their final tissue‐like appearance (Figure 1b). On the formation day (i.e., day 0), applying a pressure difference caused FN‐silk to display an initial collapse of the air bubbles entrapped within the construct. After 24 h, most air bubbles could no longer be detected and were completely gone 48 h after formation, when the final ECM‐like network was established. Pictures were taken on days 4 and 7 confirmed that the FN‐silk networks maintained their architecture over time.

Breast cancer is a heterogeneous disease with different subtypes associated with various prognoses and therapies. We chose to work with the three canonical human immortalized breast cancer cell lines SK‐BR‐3, MCF‐7, and MDA‐MB‐231, representative respectively of the main subtypes HER2‐overexpression, luminal‐like, and triple‐negative. To estimate the reproducibility among networks created with the three cell lines, we performed area measurements of the outer surfaces of the FN‐silk networks. For each cell type, 10 scaffolds were generated, and stereomicroscopic pictures of each construct were taken on day 0, after formation, and on days 2, 4, and 7 (Supplemental Figure 1a). The measurements showed that for each cell type, the size of the scaffolds remained comparable over time. MDA‐MB‐231 scaffolds (area 0.13 cm^2^ ± 0.05 cm^2^) appeared smaller than the ones generated with MCF‐7 (0.18 cm^2^ ± 0.02 cm^2^) and SK‐BR‐3 (area 0.19 cm^2^ ± 0.02 cm^2^) cells (Figure 1c). An additional analysis done to estimate the area values independently of the cell line, revealed an average scaffold area of 0.17 cm^2^, corresponding to 60% of a 96‐well plate well area (0.29 cm^2^) (Supplemental Figure 1b). The thickness of the networks was estimated to be between 150 and 200 μm by z‐stack measurements done with fluorescent microscopy, corresponding to an optimal thickness for oxygen diffusion.^23^ FN‐silk networks were completely submerged from day 2, when they were found floating in the middle of a well (Supplemental Figure 1c). Finally, to examine the interactions established by the cells in the FN‐silk network, we created scaffolds using a fluorescently‐labeled FN‐silk and let MCF‐7 grow for 7 days. Staining for cytoskeleton and nuclei of MCF‐7 highlighted grape‐like clusters of cells in close proximity to FN‐silk, demonstrating how cells establish cell–cell and cell‐matrix interactions (Figure 1d).

### 3.2 Breast cancer subtypes grow and maintain their key features in FN‐silk networks

After verifying that breast cancer cells can establish cell–cell and cell‐matrix contacts while growing within the FN‐silk networks, we investigated the viability, marker expression, and morphology of cells.

**FIGURE 2:**
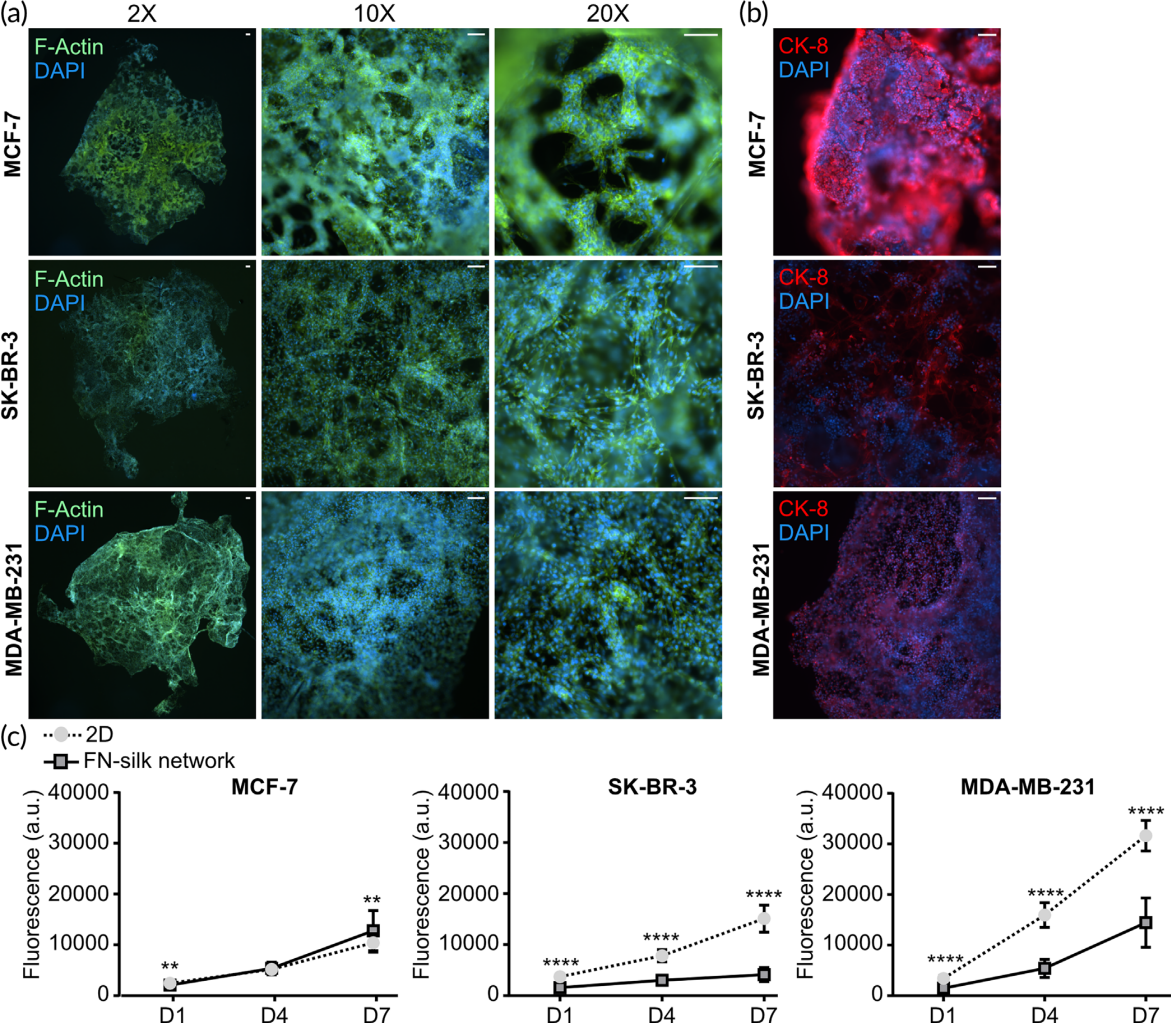
Breast cancer cells grow with a homogeneous distribution in fibronectin (FN)‐silk networks. (a) Overlay images of staining for Actin filaments (F‐Actin, green) and nuclei (DAPI, blue) were taken on MCF‐7, SK‐BR‐3, and MDA‐MB‐231 cultured in FN‐silk network for 7 days. Scale bar: 100 μm. *N* = 3 with three technical replicates each. (b) Overlay images of staining for cytokeratin 8 (CK‐8, red) and nuclei (DAPI, blue) were taken on MCF‐7, SK‐BR‐3, and MDA‐MB‐231 cultured in the FN‐silk network for 7 days. *N* = 2, with three technical replicates. Scale bar: 100 μm. (C) Graphs indicating the metabolic activity of MCF‐7, SK‐BR‐3, and MDA‐MB‐231 cells cultured in 2 dimensions (2D) or FN‐silk network. The metabolic rate was monitored over time (days 1, 4, and 7) using Alamar blue. For each cell line, three independent experiments were performed, each with 8 technical replicates per condition. **P < 0.01; ****P < 0.0001.

First, we confirmed a homogeneous cell distribution pattern in the networks by staining the cytoskeleton and nuclei of cells cultured in the scaffolds (Figure 2a). The actin filament staining highlighted how MCF‐7 formed mass‐like clusters, in contrast to SK‐BR‐3 and MDA‐MB‐231, which appeared to spread without clumping together (Figure 2a, 10×, 20× magnifications). The highly compact spatial organization of MCF‐7 was also observed by staining for keratin 8 (KRT8) (Figure 2b). We then performed the Alamar Blue assay at several time points (i.e., days 1, 4, and 7) to compare the metabolic activity of cells cultured in FN‐silk networks or on tissue‐culture treated plates. Since Alamar Blue relies on fluorescence measurement in the supernatant, no interference from the FN‐silk scaffold was detected (Supplemental Figure 1d). The data revealed that all cell lines could grow in FN‐silk networks, with the invasive cell MDA‐MB‐231 having the highest metabolic rate. SK‐BR‐3 and MDA‐MB‐231 cultured in 2D showed a higher metabolic rate than their counterpart kept in FN‐silk constructs, whereas MCF‐7 had slightly higher metabolic activity in FN‐silk networks after 7 days in culture (Figure 2c). This observation suggests a correlation between the higher metabolic rate in 3D and the ability of cells to develop complex morphologies in the silk network.

**FIGURE 3:**
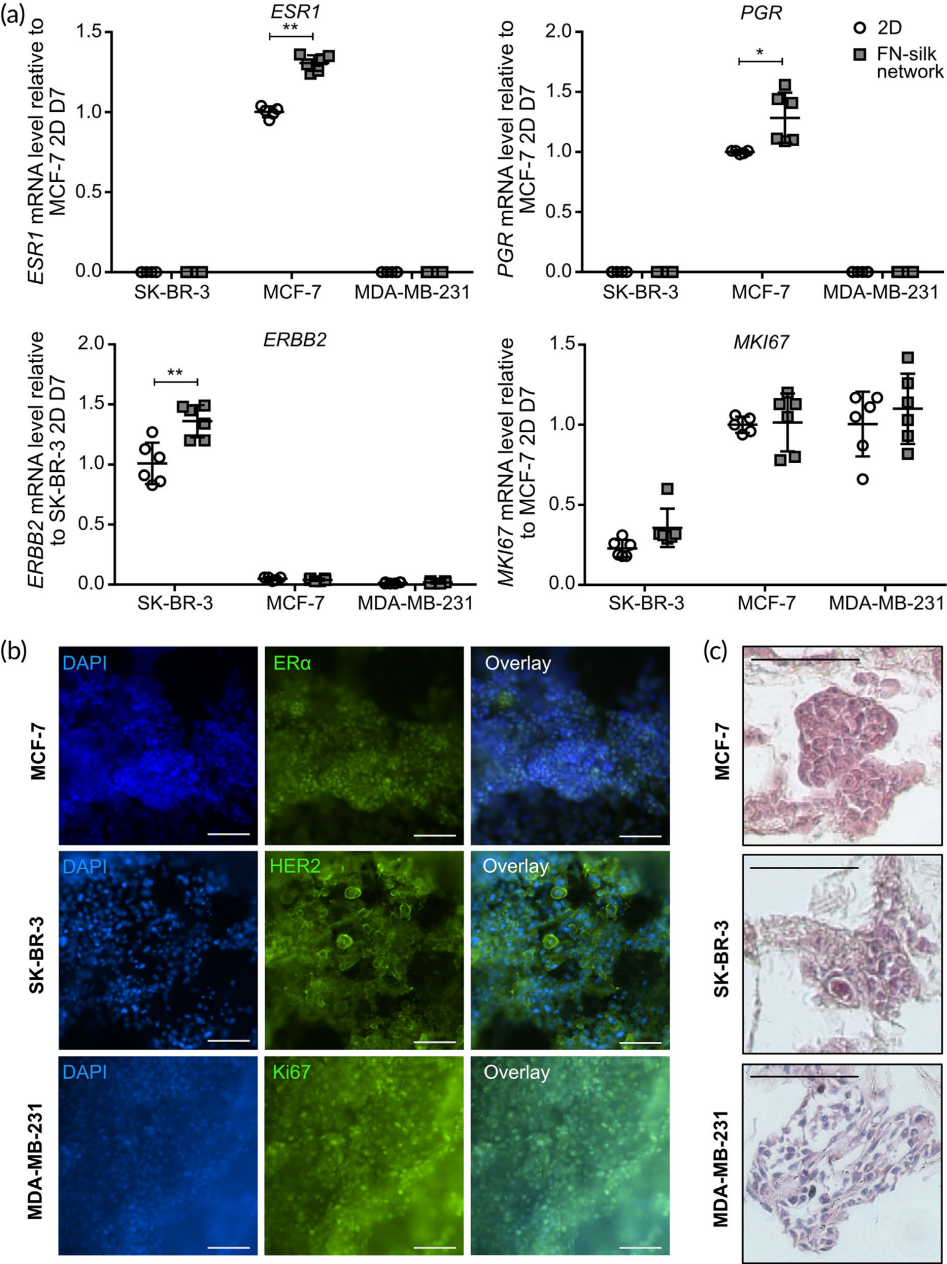
Breast cancer markers and morphological appearance of cell lines cultured in fibronectin (FN)‐silk networks. (a) Gene expression levels of the canonical breast cancer markers *ESR1, PGR, ERBB2*, and *MKI67* were measured on day 7 in SK‐BR‐3, MCF‐7, and MDA‐MB‐231 cells kept in culture in 2D or FN‐silk network. Values are represented as fold‐change of the mean ± SD (*n* = 6). Fold‐change was calculated comparing to the control MCF‐7 2D on day 7 for *ESR1*, *PGR*, and *MKI67* and to control SK‐BR‐3 2D on day 7 for *ERBB2*. *P < 0.05; **P < 0.01. (b) Immunofluorescence staining was done on MCF‐7, SK‐BR‐3, and MDA‐MB‐231 cells cultured in FN‐silk network for 7 days. MCF‐7 was stained for ERα (green), SK‐BR‐3 for HER2 (green), and MDA‐MB‐231 for Ki67. Cell nuclei were stained with DAPI (blue), and an overlay image was created. *N* = 3, with three technical replicates each. Scale bar: 100 μm. (c) Hematoxylin and eosin staining on sections of MCF‐7, SK‐BR‐3, and MDA‐MB‐231 cells cultured for 7 days in FN‐silk network. *N* = 2. Scale bar: 100 μm.

As it is crucial to maintain the features characterizing the heterogenicity of breast cancer subtypes, we next sought to assess the stability of the four classical markers estrogen receptor α (*ESR1*, ERα), progesterone receptor (*PGR*, PR), human epidermal growth factor receptor 2 (*ERBB2*, HER2) and, marker of proliferation Ki‐67 (*MKI67*, Ki67) in our FN‐silk models. The mRNA level measurement confirmed that cells maintain their characteristic features when cultured in FN‐silk networks. For instance, we detected a high expression of *ERα* and *PGR* in the luminal‐like MCF‐7, and an abundant *ERBB2* in the HER2‐overexpressing SK‐BR‐3, while these markers were absent in the triple‐negative MDA‐MB‐231. The levels of *MKI67* emphasized the high proliferation of MDA‐MB‐231 and MCF‐7 in contrast to a slower growth rate of SK‐BR‐3 (Figure 3a).

To confirm the expression at the protein level of ERα and HER2, we performed staining on whole networks after 7 days of culture. The MCF‐7 cells showed a nuclear signal for ERα, while the SK‐BR‐3 stained positively for HER2 (Figure 3b). As expected, the triple‐negative MDA‐MB‐231 was negative for both markers (Supplemental Figure 1e) while abundantly expressing Ki67 (Figure 3b). Finally, we performed hematoxylin and eosin (H&E) staining on sections of the networks (Figure 3c), detecting a distinct morphology for each cell line. For instance, MDA‐MB‐231 spread without a clear spatial reorganization, SK‐BR‐3 tended to create loose cell clusters, and MCF‐7 arranged themselves into clusters with a diameter of approximately 100 μm (Figure 3c).

### 3.3 Culture in FN‐silk networks modulates gene expression and confers signatures of invasiveness

Growth in a 3D microenvironment is known to affect the gene expression profile of cells and render them closer to actual tissues.^24^, ^25^ Processes such as adhesion, migration, angiogenesis, and EMT play a role in tumor development and invasiveness. To test if growth in FN‐silk networks affects these biological mechanisms, we performed RT‐qPCR to investigate the expression of 34 representative genes. The results revealed significant changes for 12 of the 34 targets (Supplementary Figure 2a and 2b). Growth in FN‐silk networks modulated several cell adhesion markers in MCF‐7 (i.e., *CDH2*, *JAM2*, *CLDN3*) and promoted the expression of *CD44*, a glycoprotein involved in cell–cell interactions, cell adhesion, and migration, in both MCF‐7 and SK‐BR‐3. Moreover, MDA‐MB‐231 cells grown in FN‐silk networks had high cancer stem cell markers (i.e., *NANOG*, *OCT4*, and *SOX2*), accompanied by an up‐regulation of genes associated with basement membrane degradation and invasiveness (i.e., *HIF1α*, *ITGAV*, and *MMP14*).

**FIGURE 4:**
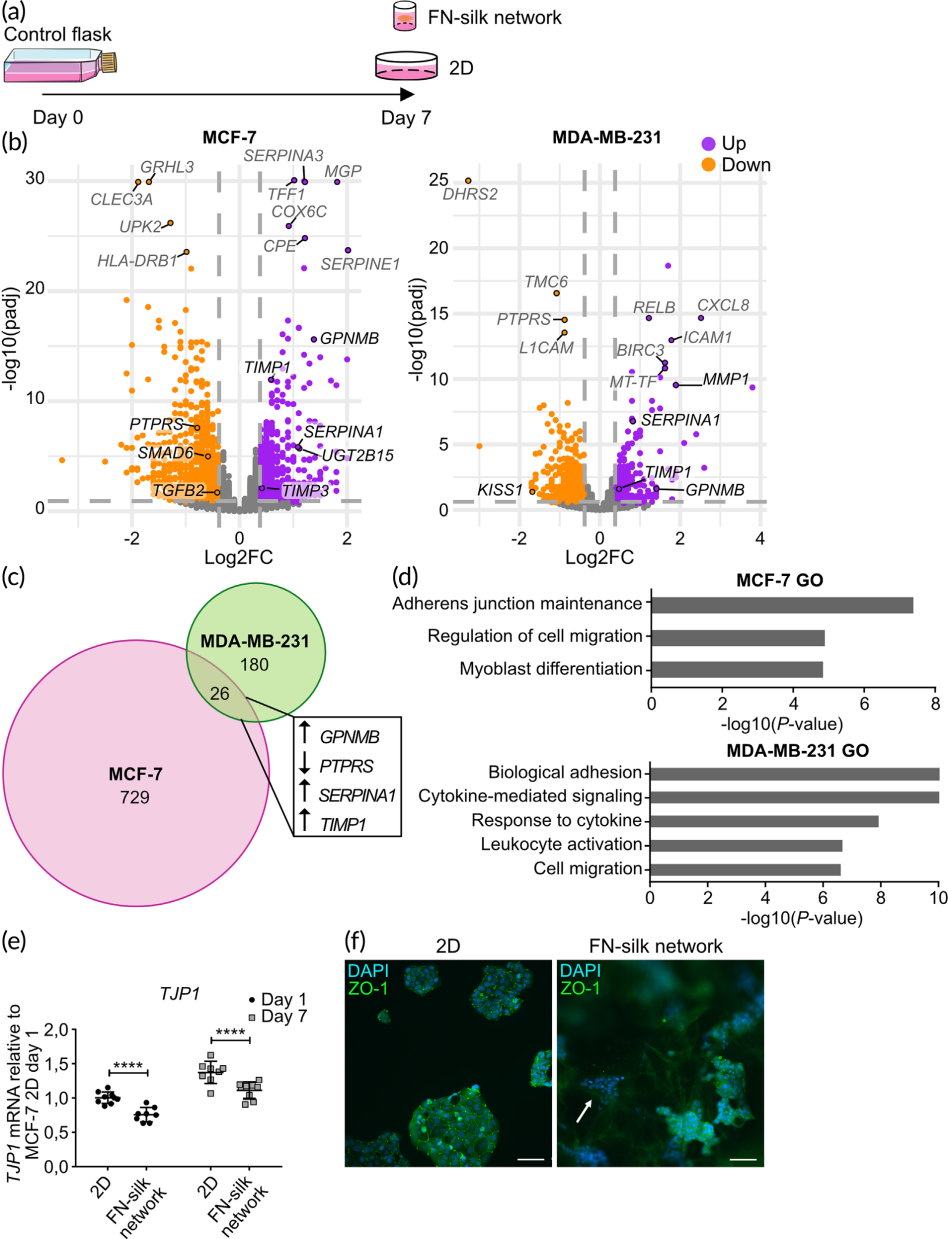
Impact of fibronectin (FN)‐silk network on global gene expression levels and ZO‐1 target validation. (a) Schematic illustrating the three conditions compared for the transcriptomic experiment, being (i) cells lysed from the control flask at day 0, (ii) cells lysed after 7 days in culture in 2D, or (iii) 7 days in culture in FN‐silk network. (b) Volcano plots of MCF‐7 and MDA‐MB‐231 transcriptomes after 7 days in culture in FN‐silk network compared to their counterparts grown in 2D. The x‐axis corresponds to the log2FC, and the y‐axis to the negative log10 of the adjusted *P*‐value. Transcripts with an adjusted *P‐*value <0.05 are shown in purple if log2FC ≥0.38 and in orange if log2FC ≤ −0.38. The threshold values are represented with thicker dark gray lines. Transcripts not meeting the criteria are depicted in gray. The most significantly regulated genes are indicated in dark gray, whereas selected targets relevant to the pathophysiology of cancer are indicated in black. (c) Proportional Venn diagram representing the number of significantly DEGs due to culturing of MCF‐7 (pink) and MDA‐MB‐231 (green) in FN‐silk network. 26 transcripts are commonly regulated in both cell lines. Among these, genes with key roles in breast cancer are indicated (i.e., *GPNMB*, *PTPRS, SERPINA1, TIMP1*). (d) Gene enrichment analysis of the FN‐silk network signature in MDA‐MB‐231 and MCF‐7. Web‐Gestalt was used to explore the gene ontology terms associated with the FN‐silk network signature. The bars represent the negative log10 (*P*‐value) of enriched terms, indicating the significance of the association between the gene list and an indicated ontology term. (e) mRNA levels of tight junction protein‐1 (*TJP1*), a significantly decreased gene in MCF‐7 grown in FN‐silk network compared to cells kept in 2D. Values are represented as fold‐change of the mean ± SD (*n* = 3 with three technical replicates). Fold‐change was calculated compared to the control MCF‐7 2D on day 1. For the statistical analysis, 2‐way ANOVA was performed, ****P < 0.0001. (f) *TJP1‐*encoded protein zonula occludens‐1 (ZO‐1, green) and nuclei (DAPI, blue) were stained in MCF‐7 cultured in 2D or FN‐silk network for 7 days. The arrow points to a cell cluster in which ZO‐1 was not detected. A representative overlay image is shown (*n* = 2, with three technical replicates). Scale bar: 100 μm.

To obtain the complete global transcriptome changes driven by culture in FN‐silk networks, we decided to use RNA‐sequencing (RNA‐seq). For this experiment, we focused on the two cell lines MCF‐7 and MDA‐MB‐231. This choice was motivated by the observed robust metabolic rate and the high number of modulated transcripts associated with culture of these two lines in FN‐silk. For the RNA‐seq, we compared three conditions, being (1) cells directly lysed after trypsinization from the flask at day zero, referred to as the control flask, (2) cells cultured for 7 days in FN‐silk network, and (3) cells that were grown for 7 days on a flat tissue‐culture treated plate, as illustrated in the cartoon of Figure 4a.

A principal component analysis (PCA) was conducted to detect eventual outliers and to evaluate the effect of cell type (i.e., MCF‐7 and MDA‐MB‐231), culturing condition (i.e., 2D or 3D), and time (i.e., day 0 and 7) on the results. The first exploratory PCA analysis took into account the results from both cell lines and revealed the cell type as the factor accounting for 99% of the variance (Supplemental Figure 3a). This data confirmed a major difference between the two breast cancer subtypes. Considering such observation, we processed the datasets generated using the two cell lines independently. These PCAs showed that for both MDA‐MB‐231 and MCF‐7, the cultivation of cells in FN‐silk network caused the greatest variance, while time had only a minor effect (Supplemental Figure 3b, 3c). Additionally, we observed a greater effect caused by FN‐silk for MCF‐7 (62%) than for MDA‐MB‐231 (46%).

Culture in FN‐silk network led to 755 DEGs in MCF‐7 and 206 in MDA‐MB‐231, which had a similar distribution between up‐ and down‐regulated, as shown by the volcano plots (Figure 4b, full list of DEGs is available in Supplemental Table 1). While 26 DEGs were shared between the two cell lines (Figure 4c), only 17 responded in a similar fashion. Among these 17 genes, there were genes involved in cancer progression, for instance, the down‐regulated tumor suppressor *PTPRS* ^26^ and the up‐regulated *SERPINA1* whose overexpression was reported to increase invasiveness,^27^ *GPNMB*, which induces stem‐cell‐like properties in cancer cells,^28^ and *TIMP1* a promoter of tumorigenesis.^29^ These 17 genes were also tested in SK‐BR‐3 cells, showing a conserved decrease in *AP1G2* and *PTPRS*, as well as an increase in *TIMP1* (Supplementary Figure 4a).

Gene ontology analyses revealed cell‐specific signatures, such as inflammation in MDA‐MB‐231 and myoblast differentiation in MCF‐7, as well as common biological processes of migration and adhesion. Additionally, to investigate whether the genes modulated by culture in FN‐silk networks had a profile relevant to breast cancer biology, we performed a GSEA against a functional database built on the genes of the Nanostring nCounter® Breast Cancer 360 panel, which includes 758 genes that cover established breast cancer diagnostic and research signatures (e.g., PAM50) as well as key pathways of the tumor and its microenvironment. This data exploration showed a significant enrichment score for the genes in MCF‐7, highlighting that cultivation in FN‐silk networks affects the expression of targets important for breast cancer biology (Supplementary Figure 4b).

To investigate which TFs were mediating the observed changes, we performed two bioinformatic predictions. The first was done using i‐*cis* Target, an integrative method that identifies shared regulatory regions in the promoters of sets of DEGs.^21^ The results revealed RELA/P65, a subunit of the NFKβ inflammatory complex, as the main responsible for the changes observed in MDA‐MB‐231, corroborating the gene ontology results and suggesting a higher inflammatory response in MDA‐MB‐231 cultured in FN‐silk networks than in 2D. In MCF‐7, the promoters of the DEGs were mostly enriched for two TFs, being (1) TF AP‐2 alpha (TFAP2A), which is known to play a role in EMT,^30^ and (2) the TEA domain family members (TEADs), who support cancer progression by promoting genes related to proliferation.^31^

For the second bioinformatic prediction, we took advantage of a complementary approach based on prior knowledge of expected effects between transcriptional regulators, not exclusively TFs, and their target genes stored in the Ingenuity® Knowledge Base.^22^ While the results confirmed NFKβ as the most activated regulator responsible for the changes of MDA‐MB‐231, they highlighted nuclear protein 1 (NUPR1), previously associated with breast cancer metastasis, as the most significantly activated regulator in MCF‐7 (full list available in Table 2). In addition to being predicted as activated, *NUPR1* also had an increased mRNA level in MCF‐7 grown in FN‐silk. NUPR1 has emerged as a repressor of ferroptosis, a type of iron‐dependent regulated cell death.^32^ Since a recent study suggested a new tumor‐mediated control of iron permitted by the 3D tumor architecture,^33^ we explored the DEGs in MCF‐7 for targets related to iron storage. We found that culture in FN‐silk increased both light (*FTL*) and heavy (*FTH1*) chains of Ferritin, the major intracellular iron storage protein.

**TABLE 2:** Transcriptional regulators. List of the upstream regulators predicted to mediate the gene expression changes observed in MCF‐7 and MDA‐MB‐231 following culture in FN‐silk networks.

(a)			
MCF‐7			
Transcriptional regulator	Predicted state	Activation z‐score	*P*‐value
NUPR1	Activated	3.3	0.0175
ESR1	Activated	3.1	4.1E‐0.5
WBP2	Activated	2.4	0.0204
TCF4	Activated	2.3	5.23E‐0.4
RAD21	Inhibited	−2.0	5.87E‐0.4
NANOG	Inhibited	−2.1	7.35E‐0.3

(b)			
MDA‐MB‐231			
Transcriptional regulator	Predicted state	Activation *z*‐score	*P*‐value
RELA/NFKB	Activated	2.9	4.5E‐11
SREBF1	Activated	2.9	1.5E‐05
NFAT5	Activated	2.8	1.0E‐05
EGR1	Activated	2.8	1.72E‐0.5
NCOA2	Activated	2.4	8.51E‐0.4
ECSIT	Activated	2.4	1.10E‐0.7
ATF4	Activated	2.4	7.39E‐0.4
STAT1	Activated	2.4	1.25E‐0.2
BHLHE40	Activated	2.4	5.8E‐04
HMGB1	Activated	2.4	1.73E‐0.7
FOXL2	Activated	2.2	2.71E‐0.4
EZH2	Activated	2.2	6.13E‐0.5
GFI1	Inhibited	−2.0	1.7E‐04
BCL3	Inhibited	−2.3	6.3E‐06
SATB1	Inhibited	−2.4	3.0E‐03
ZFP36	Inhibited	−2.4	1.7E‐06

Tight junctions have a vital role in maintaining cell‐to‐cell integrity, and the loss of cell cohesion can lead to invasion and, thus, metastasis of cancer cells.^34^ Tight junction protein 1 (*TJP1*) is considered a tumor suppressor, and its downregulation has been associated with invasive features of cancer. We identified *TJP1* among the adhesion genes with decreased expression in MCF‐7 grown in FN‐silk networks. We confirmed that the decrease of *TJP1* can be detected already after 24 h and is maintained over time (Figure 4e). Afterward, we performed staining for the protein coded by *TJP1*, zonula occludens‐1 (ZO‐1), revealing that MCF‐7 cultured in 2D formed monolayer clusters in which ZO‐1 was always detectable, whereas in FN‐silk networks some clusters did not express ZO‐1 (Figure 4f and Supplementary Figure 4c).

### 3.4 FN‐silk networks as a flexible system to grow physiologically relevant cancer cells

In the last part of the study, we investigated if it is possible to use the FN‐silk networks for the culture of novel breast cancer cell lines as well as primary cells obtained from fresh human breast tumors.

First, we tested if FN‐silk networks could support the growth of the Wood and PB cells, two lines with phenotypic properties which faithfully reproduce the original cancer tissues.^35^ These cells complement canonical immortalized cells by better representing the variability observed among cancer patients. The comparison of breast cancer markers between Wood, PB, and the canonical SK‐BR‐3, MCF‐7, and MDA‐MB‐231, showed how the ERα + Wood cells have much lower levels of ERα than the classically used MCF‐7, whereas PB resembles a triple‐negative subtype, but with lower proliferation rate than the MDA‐MB‐231 (Supplementary Figure 5).

**FIGURE 5:**
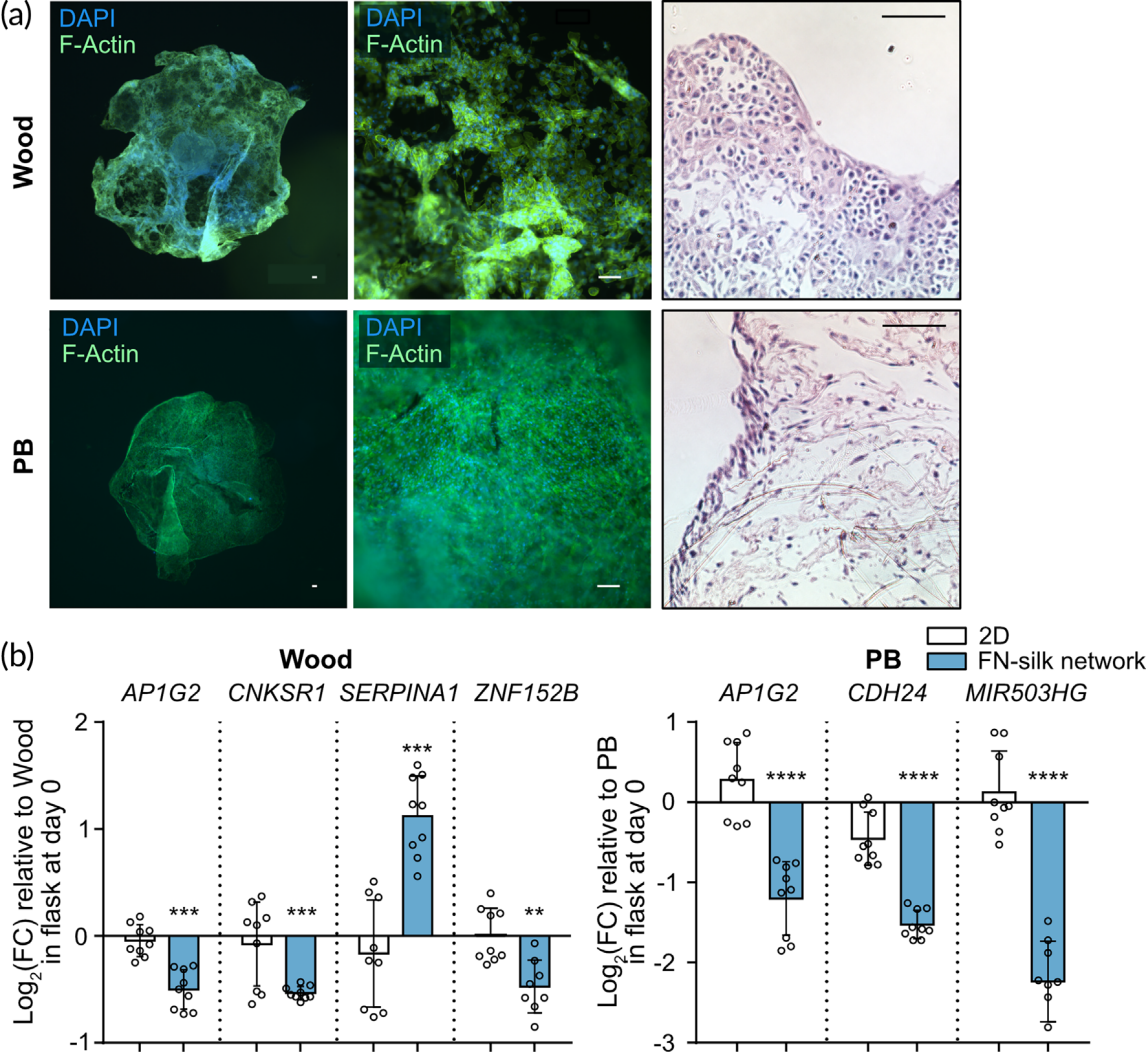
Wood and PB breast cancer cells grow in fibronectin (FN)‐silk networks. (a) Wood and PB breast cancer cells cultured for 7 days in the FN‐silk network were stained for actin filaments (F‐actin, green) and nuclei (DAPI, blue). Overlay images of pictures taken with 2× and 10× magnification are shown for both cell lines (*n* = 2, 3 technical replicates). Scale bar: 100 μm. The figures on the right side of the panel show hematoxylin and eosin staining on sections of Wood and PB cells cultured for 7 days in FN‐silk network, 10× magnification (*n* = 2, 3 technical replicates). Scale bar: 100 μm. (b) Transcript levels of genes commonly regulated by growth on the FN‐silk network were measured on Wood and PB harvested from the control flask on day 0 or from cells grown in 2D or FN‐silk network for 7 days. Values are represented as log2 fold‐change of the mean ± SD (*n* = 3, 3 technical replicates). Fold‐change was calculated compared to the control flask on day 0. For the statistical analysis, 2‐way ANOVA was done. *P < 0.05; **P < 0.01; ***P < 0.001; ****P < 0.0001.

Staining for the cytoskeleton protein F‐actin demonstrated that wood and PB cells could homogeneously spread in FN‐silk networks (Figure 5a). The results also indicated a cell‐specific morphology. For instance, Wood cells tended to reorganize themselves into clusters, and PB cells created a more continuous layer of cells. The distinctive cellular architecture was confirmed by H&E staining. Additionally, we tested if any of the 17 commonly regulated genes in MCF‐7 and MDA‐MB‐231 were modulated in Wood and PB. The results showed that for Wood cells, culture in FN‐silk networks caused a decrease in *AP1G2*, *CNKSR1*, and *ZNF152B*, as well as increased *SERPINA1*. For PB cells, we detected a significant down‐regulation of *AP1G2*, *CDH24*, and *MIR503HG* (Figure 5b).

**FIGURE 6:**
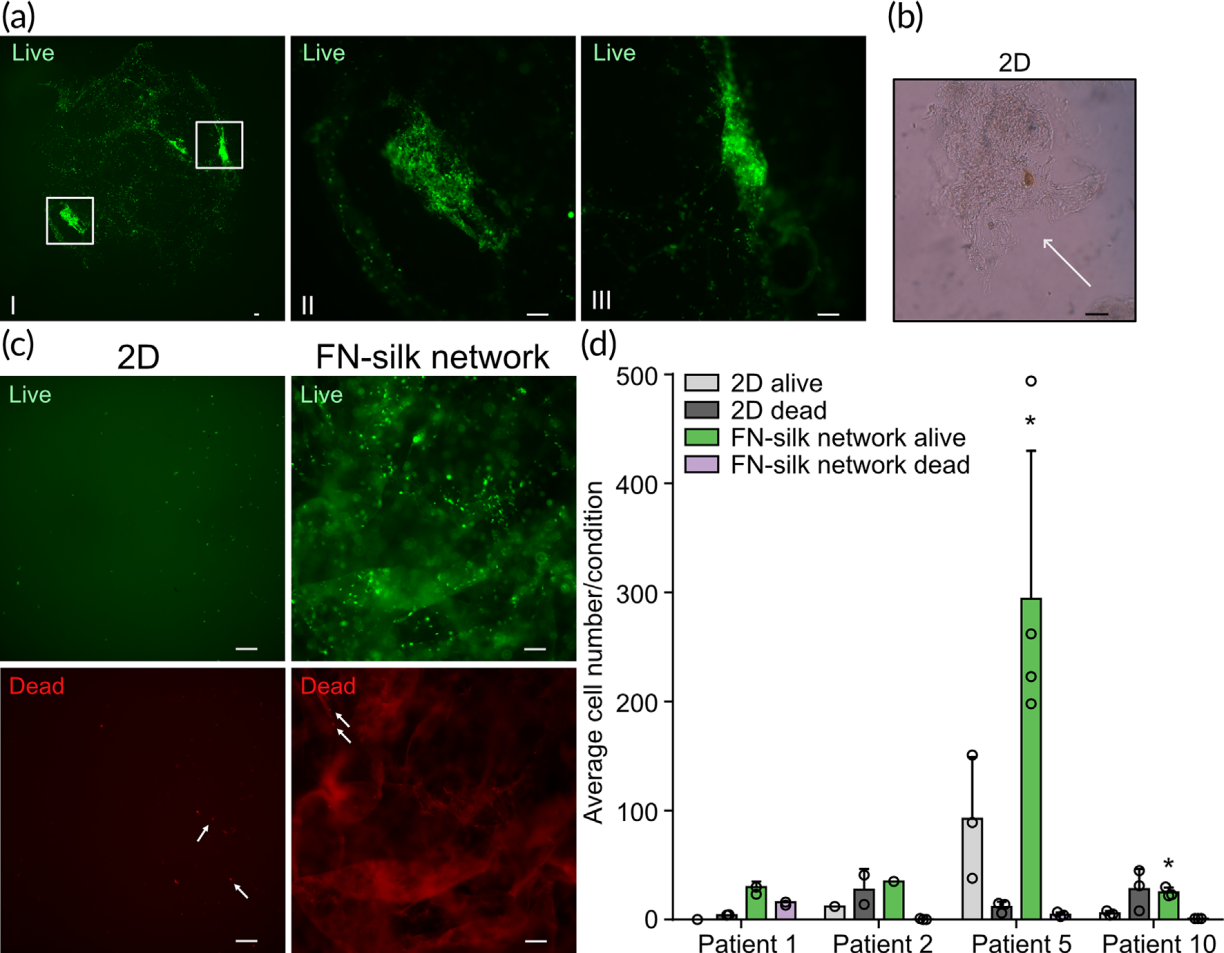
Fibronectin (FN)‐silk network promotes viability of cells dissociated from patients tumors. Live/dead assay is done on FN‐silk networks containing cells obtained from breast cancer tissue and cultured for 7 days. (a) Live channel image of a FN‐silk network scaffold generated using cells from patient 5. Picture (I) was taken with a 2× magnification, while (II) and (III), which show areas with abundant cell clusters, were obtained with a 10× magnification. Scale bar: 100 μm. (b) Brightfield image of a cell cluster floating in a 2D well, following the dissociation of the original tumor material (*n* = 1). 10× magnification, scale bar: 79 μm. (c) Representative pictures of live (green) and dead (red) staining of cells grown in 2D and FN‐silk network for 7 days, 10× magnification. The white arrows point to examples of dead cells (*n* = 4). Scale bar: 100 μm. (d) Quantification of alive and dead cells from four biological replicates. No pink bar is visible for the biopsies corresponding to patients 2 and 10 because no dead cells were observed in the FN‐silk network condition. For each condition of each biological replicate, pictures from three areas of the sample were considered when possible. For the statistical analysis, 1‐way ANOVA was done. *P < 0.05.

Finally, we examined the potential of FN‐silk networks to grow primary cells obtained via superficial scraping of tumor material following surgical removal.^36^ For each sample, the tissue was subjected to enzymatic dissociation. The cell suspension was then seeded on tissue‐culture‐treated 96‐wells or mixed with FN‐silk. To compare the viability of the patient‐derived cells in 2D and FN‐silk networks, live/dead staining was performed after 7 days of culture. The data revealed alive cells across the FN‐silk network (Figure 6a‐I). Areas with a high density of cells were also detected (Figure 6a‐II, a‐III), suggesting that even incompletely dissociated tissue was kept alive in the network. Such observation was in contrast with what was seen in the 2D control, where floating cell clusters were visible directly after seeding (Figure 6b) but lost during the routinely performed medium change.

After the dissociation step, we generally found a low number of cells, with the enzymatic dissociation leading to approximately 20,000 cells per sample. Because of the low cell number constraint, we were able to perform quantifications of live and dead cells for only four of the 10 biopsies. This was done in ImageJ by counting the number of cells in three different regions of each sample from the live (green) and dead (red) channels (Figure 6c). Notably, FN‐silk has an autofluorescence emission in the red channel, which causes a red background signal, examples of dead cells in FN‐silk network are indicated with white arrows (Figure 6c). The results of this quantification revealed a higher number of cells in FN‐silk than in 2D, with more dead cells visible in 2D than in FN‐silk networks (Figure 6d). A trend of higher viability in FN‐silk network than 2D was observed for patient 1 and 2 but due to insufficient data points, for them, we could not perform statistical analysis. The statistical analysis could only be done for samples from patients 5 and 10. Patient 5 was characterized by the highest cell number after dissociation, reflected by the high general number of viable cells after 7 days in culture. These results indicate that the FN‐silk scaffold can support patient‐derived cell growth more efficiently than conventional 2D culture.

## 4 DISCUSSION

Our findings demonstrate the possibility of growing immortalized cells as well as primary cells obtained from the superficial scraping of fresh tumors in floating networks made of recombinant spider silk protein 4RepCT genetically functionalized with the cell attachment motif from FN (FN‐silk). Such a model shows an advantage over gel‐based and scaffold‐free alternatives since it recapitulates cell–cell and cell‐ECM interactions. Existing scaffold‐based options often suffer from batch‐to‐batch variability^12^ and low seeding efficiency.^37^ Silk fibroin obtained from *Bombyx mori* silkworm cocoons has previously been used to form scaffolds to support the growth of cancer cells.^38^, ^39^ Compared to such studies, the herein‐described FN‐silk has the advantage of a completely controlled composition and a scaffold formation that can be carried out under physiological conditions. These strengths are complemented by optimal seeding distribution and cell attachment thanks to the properties of FN‐silk.^15^, ^16^, ^18^

Previously, we showed that pipetting air bubbles into a solution of FN‐silk leads to the self‐assembly of FN‐silk sheets around each air bubble, which, when bursting, yields a network where cells can stretch out and proliferate.^17^ These silk networks had been prepared anchored to the bottom of a tissue‐culture plate. We here adapted the published protocol introducing three main novelties to the method, being: (a) the use of immortalized and primary human breast cancer cells, (b) a step for fast removal of air bubbles from the scaffold to better mimic physiological conditions, and (c) the culturing of a floating network in which all the sides are equally exposed to the medium. During the first 24 h, FN‐silk networks float with those parts containing air bubbles reaching the top part interface of the liquid, where they may experience high oxygenation and later position themselves towards the mid‐height of the well. This behavior, together with their thickness (i.e., 150–200 μm) and fibrous structure, ensures oxygen and nutrient distribution to all regions of the construct for most of the culturing time.

Several publications have reported that cells proliferate slower in 3D than 2D.^40^ In our study, metabolic activity measurements, commonly used to infer the proliferation rate of cells, only partly supported the existing literature. For instance, while the results obtained with SK‐BR‐3 and MDA‐MB‐231 confirmed previous data, MCF‐7 had higher metabolic activity in FN‐silk than 2D. The precise mechanism leading to this difference is unclear. However, we speculate it may be related to the cell‐specific modality of reorganizing into space. As we estimated the area of each network to be about 40% smaller than the one of a 96‐well, such a factor might have limited the proliferation of cells spreading without complex cell–cell morphologies (i.e., SK‐BR‐3 and MDA‐MB‐231) and, on the contrary, favored MCF‐7, which created multiple compact cell clusters into the FN‐silk construct.

Breast cancer patients are routinely stratified based on their distinct expression of markers. Since prognoses and treatments are tailored for each subtype, representative cell lines must recapitulate the features of the various types. We showed that culture in the FN‐silk network does not lead to a loss in breast cancer markers. Additionally, we proved how each cell line developed a distinctive morphology when cultured in FN‐silk, consistent with what was observed in Matrigel‐based models.^41^

Studies using spheroids and gel‐based scaffolds have demonstrated that growth in environments more complex than classical flat surfaces influences gene and protein expression.^24^ Herein, we investigated the transcriptional changes occurring when cancer cells are maintained in silk‐based networks. Our sequencing data revealed cell‐type‐specific gene regulation driven by these culture conditions. While the affected genes largely differed among the two cell lines, the analysis revealed that many were related to cell adhesion and migration. Both MCF‐7 and MDA‐MB‐231 had upregulated expression of genes promoting invasiveness (*SERPINA1* ^27^), ECM remodeling (*TIMP1* ^29^), and tumorigenicity (*GPNMB* ^28^). N‐cadherin, a protein mediating cell–cell contact, often upregulated in aggressive cancers, and reported to promote migratory behaviors in a collagen‐based 3D model,^42^ was highly expressed in cells cultured in FN‐silk. Growth in the FN‐silk network led to a decrease in ZO‐1, a protein that anchors tight junctions to the actin filaments of the cytoskeleton and whose down‐regulation correlates with invasiveness.^43^ Overall, these results suggest that our scaffolds offer an environment where tumor cells can attach, modify, and migrate through the remodeled matrix.

Some of the changes observed in the triple‐negative cells comprised (a) an increase in cancer stem cell markers, similar to what was found in collagen and spheroid models,^44^ (b) higher expression of the hypoxia‐inducible factor *HIF1α*, which promotes angiogenesis and capability to metastasize,^45^ and (c) an NFκB‐mediated inflammatory signature. NFκB, the main driver of cytokine expression in immune cells, was reported to promote the secretion of pro‐inflammatory cytokines and contribute to aggressive tumoral features in triple‐negative cells.^46^, ^47^ Our data suggest that culture in FN‐silk could trigger a high level of pro‐inflammatory signaling. Since NFκB inhibitors are emerging as drugs to treat triple‐negative cancers, our model could be used to study such medications.

Most of the literature comparing gene expression changes in 2D as opposed to 3D models has focused on a small set of genes. To our knowledge, only two studies considered the global transcriptional changes of MCF‐7 grown in 3D, thus being somewhat comparable to our investigation. Blanchette‐Farra's research analyzed transcriptomic variations in MCF‐7 cultured in three spheroid models.^33^ They generated spheroids using; (a) plates coated with poly(2‐hydroxyethyl methacrylate), (b) ultra‐low attachment plates, and (c) methylcellulose as an aggregating agent. Spheroids from these conditions had similar transcriptional changes (represented by 770 DEGs). In another study, Wulftange et al. maintained MCF‐7 in Matrigel mixed with FN solution and identified 3156 DEGs.^48^ The signatures from the two published datasets shared 240 common DEGs. A similar low overlap is also present when we consider the FN‐silk‐dependent genes in MCF‐7, leading to 74 genes in common with the spheroids^33^ and 208 with the Matrigel model.^48^ Our study and the previous two shared only 24 genes. Among these are (a) an increase of *UGT2B15*, which detoxifies drugs and has been shown to influence carcinogenesis in hormone‐dependent cancers,^49^ and (b) a higher level of *FTL*, the light subunit of ferritin, a major intracellular iron storage protein. This comparison highlights the limited reproducibility among independent studies, which could be partially reduced using components with a highly defined composition and low batch‐to‐batch variability, such as FN‐silk. Nevertheless, it also suggests the presence of conserved mechanisms triggered by three‐dimensional settings, such as higher detoxification ability and, thus, drug resistance as well as modulation of iron‐storage proteins.

Additionally, the study from Blanchette‐Farra^33^ suggested a new tumor architecture‐dependent increased ability of iron storage in 3D cultures, which reduces ferroptosis, an iron‐regulated cell death. Consistently with their study, we found both light and heavy subunits of ferritin up‐regulated in MCF‐7 grown in FN‐silk. Moreover, we observed an increased mRNA level of *NUPR1*, a TF that can repress ferroptosis,^32^ and bioinformatically predicted its activation in MCF‐7 grown in FN‐silk. It is tempting to speculate that our model favors an increase in intracellular iron storage via ferritin that, together with the activated ferroptosis repressor NUPR1, may decrease ferroptosis and render MCF‐7 cells more resistant.

While we could demonstrate that gene expression changes relevant to tumor development and aggressiveness are modulated in FN‐silk networks, the precise mechanisms involved are still elusive. Future investigations should be performed to establish the contribution of the three‐dimensional nature and of the RGD motif from FN to the observed transcriptional changes.

Recapitulating the tumor microenvironment by mimicking the ECM and including cells representing tumor heterogeneity is key for creating in vitro models. FN‐silk networks offer a fibrous structure that could support the novel Wood and PB cells, which phenocopy the original primary tumors thanks to a defined and optimized medium.^35^ The protocol for FN‐silk network formation was proved to be flexible and capable of sustaining cell viability even in the case of minimal initial material with slowly proliferating cells, such as the one obtained by superficial scraping of breast cancers.^36^ This is valuable since low cell number and slow proliferation are a challenge for spheroids formation.^50^ The detection of live cell clusters also confirmed the high adaptability of our 3D model. For instance, the enzymatic dissociation step had to be performed quickly to avoid harming the patient‐derived cells. This technical limitation resulted in a suboptimal dissociation, as indicated by the presence of cell clusters lost during media exchange in 2D plates but maintained in FN‐silk networks. An additional challenge was given by the high heterogeneity in starting material quality, which reflects the biological variability of patient‐derived material and the difficulty of preserving such delicate samples. The FN‐silk scaffold's exquisite ability to favor cell attachment led to generally higher viability of cells obtained via superficial scraping of breast tumor, offering encouraging proof‐of‐concept results. Future studies should investigate whether the ability of FN‐silk networks to support cells independently of their proliferation ability and morphology could allow to co‐culture different cell types and recreate the original tumor niche.

## 5 CONCLUSION

In this study, we described a new method to generate floating networks of breast cancer based on FN‐silk. We demonstrated that our model allows to culture well‐established and novel cell lines. We performed the first investigation of transcriptomic changes driven by culture in FN‐silk networks for the two highly used MCF‐7 and MDA‐MB‐231, providing a solid foundation for future studies aiming to adopt this model. The data highlighted how culture in FN‐silk networks modulates adhesion, migration, and the expression of genes that play a role in tumor development. Overall, suggesting that our model recapitulates the tumoral features more faithfully than 2D. We also proved that patient‐derived cells can be cultured in FN‐silk networks, holding promise for the recreation of miniaturized tumoral niches.

## AUTHOR CONTRIBUTIONS

**Caterina Collodet:** Conceptualization (equal); data curation (lead); formal analysis (lead); funding acquisition (equal); investigation (lead); methodology (equal); supervision (equal); validation (lead); visualization (lead); writing – original draft (lead). **Kelly Blust:** Data curation (supporting); formal analysis (equal); visualization (equal); writing – review and editing (supporting). **Savvini Gkouma:** Investigation (equal); writing – review and editing (supporting). **Emmy Ståhl:** Formal analysis (equal); investigation (equal); visualization (equal). **Xinsong Chen:** Methodology (supporting); resources (equal); writing – review and editing (supporting). **Johan Hartman:** Methodology (supporting); resources (equal). **My Hedhammar:** Conceptualization (lead); formal analysis (equal); funding acquisition (lead); methodology (equal); project administration (lead); resources (equal); supervision (equal); visualization (equal); writing – original draft (equal); writing – review and editing (lead).

## CONFLICT OF INTEREST STATEMENT

My Hedhammar has shares in Spiber Technologies AB, a company that aims to commercialize recombinant spider silk.

### PEER REVIEW

The peer review history for this article is available at https://www.webofscience.com/api/gateway/wos/peer-review/10.1002/btm2.10537.

## Supporting information


**Supplementary Figure 1.** Additional characterization of FN‐silk network constructs and negative control staining for ERα and HER2. (a) Representative stereo microscope pictures of the full FN‐silk networks were taken on days 0, 2, 4, and 7. The white line indicates the border of the FN‐silk network, which was drawn to determine the area of each scaffold (*n* = 3, with 9 technical replicates). Scale bar: 500 μm. (b) Grouped plots display the size of the FN‐silk network areas as a percentage compared to the surface of a 96‐well plate well. Mean ± SD, with individual data points shown (*n* = 3 with 9 technical replicates per independent experiment). (c) Pictures of two wells from a 96‐wp in which FN‐silk networks (day 2) are floating in the mid‐height part of the well. The white arrows point to the floaters. (d) Graph showing the fluorescence detected after incubation with 10% Alamar Blue medium in FN‐silk network with cells (3 technical replicates) as compared with control without cells (8 replicates) and empty wells (3 replicates). (e) Immunofluorescence staining for ERα, (green), HER2 (green), and nuclei (DAPI, blue) in MDA‐MB‐231 cells cultured in the FN‐silk network for 7 days (*n* = 2, 3 technical replicates). Single channels and overlay images are shown. Scale bar: 100 μm.
**Supplementary Figure 2.** RT‐qPCR analysis of gene expression changes driven by FN‐silk network. (a) Venn diagram representing the three biological processes, namely angiogenesis, adhesion and migration, and epithelial to mesenchymal transition (EMT), for which significantly regulated genes were identified. (b) Expression levels of the genes differentially expressed in MCF‐7, SK‐BR‐3, and MDA‐MB‐231 due to cultivation in FN‐silk network for 7 days. Values are represented as log2 fold‐change of the mean ± SD (*n* = 2, 3 technical replicates). Fold‐changes were calculated compared to the control 2D on day 1. For the statistical analysis, 2‐way ANOVA with Sidak correction was done. *P < 0.05; **P < 0.01; ***P < 0.001; ****P < 0.0001.
**Supplementary Figure 3.** Principal component analysis (PCA) score plots. (a) PCA score plot based on the global gene expression data generated from comparing MCF‐7 and MDA‐MB‐231 harvested at day 0 from the flask or at day 7 after culture in 2D or FN‐silk network. The first two principal components (PC1 and PC2) explain 99% and 1% of the total variance in the data and correspond to cell type and FN‐silk network effect on MCF‐7, respectively. (b) PCA score plot was obtained from the gene expression dataset solely of MDA‐MB‐231 samples. The FN‐silk network after 7 days in culture is responsible for 46% of the total variance in the data, as shown by PC1. (c) PCA score plot representing the MCF‐7 subset of samples. The FN‐silk network effect after 7 days in culture accounts for 62% of the total variance in the data. Samples are depicted as indicated in the figure. *N* = 3 for each cell line.
**Supplementary Figure 4.** FN‐silk network targets, further investigation at transcriptional and protein levels. (a) mRNA levels of genes commonly regulated by growth on FN‐silk network were compared in SK‐BR‐3 grown in 2D or FN‐silk network for 7 days versus at day 1 in 2D. Values are represented as log2 fold‐change of the mean ± SD (*n* = 2, three technical replicates). For the statistical analysis a t‐test was done. *P < 0.05; **P < 001; ****P < 0.0001. (b) Gene set enrichment analysis (GSEA)‐enrichment plot revealing a significant enrichment score when comparing the FN‐silk network signature in MCF‐7 with the gene panel of Nanostring Breast Cancer 360. (c) Staining of ZO‐1 and nuclei done on MCF‐7 cultured in 2D or FN‐silk network for 7 days. Single channels are shown. *n =* 2, with three technical replicates. Scale bar: 100 μm.
**Supplementary Figure 5.** Breast cancer markers in Wood and PB cells. Gene expression levels of the canonical breast cancer markers *ESR1, PGR, ERBB2*, and *MKI67* were measured in SK‐BR‐3, MCF‐7, MDA‐MB‐231, Wood, and PB cells kept in culture in 2D. Values are represented as fold‐change of the mean ± SD (*n* = 3). Fold‐change was calculated comparing MCF‐7 for *ESR1* and *PGR*, SK‐BR‐3 for *ERBB2*, and MDA‐MB‐231 for *MKI67*.

**Supplementary Table 1.** List of differentially expressed genes in response to culture in FN‐silk network.

## Data Availability

The RNA‐seq data that support the findings of this study are openly available in NCBI's Gene Expression Omnibus at (https://www.ncbi.nlm.nih.gov/geo/query/acc.cgi?acc=GSE209570), reference number (GSE209570). Additional data are available as supplementary materials published together with this article.
