# Supplementary material for: Development and characterization of a recombinant silk network for 3D culture of immortalized and fresh tumor‐derived breast cancer cells
Source: Bioeng Transl Med. 2023 May 11;8(5):e10537. doi: 10.1002/btm2.10537 (PMC10487315; doi:10.1002/btm2.10537)
Supplement: Supplementary file 1 — Supplementary Figure 1. Additional characterization of FN‐silk network constructs and negative control staining for ERα and HER2. (a) Representative stereo microscope pictures of the full FN‐silk networks were taken on days 0, 2, 4, and 7. The white line indicates the border of the FN‐silk network, which was drawn to determine the area of each scaffold (n = 3, with 9 technical replicates). Scale bar: 500 μm. (b) Grouped plots display the size of the FN‐silk network areas as a percentage compared to the surface of a 96‐well plate well. Mean ± SD, with individual data points shown (n = 3 with 9 technical replicates per independent experiment). (c) Pictures of two wells from a 96‐wp in which FN‐silk networks (day 2) are floating in the mid‐height part of the well. The white arrows point to the floaters. (d) Graph showing the fluorescence detected after incubation with 10% Alamar Blue medium in FN‐silk network with cells (3 technical replicates) as compared with control without cells (8 replicates) and empty wells (3 replicates). (e) Immunofluorescence staining for ERα, (green), HER2 (green), and nuclei (DAPI, blue) in MDA‐MB‐231 cells cultured in the FN‐silk network for 7 days (n = 2, 3 technical replicates). Single channels and overlay images are shown. Scale bar: 100 μm. Supplementary Figure 2. RT‐qPCR analysis of gene expression changes driven by FN‐silk network. (a) Venn diagram representing the three biological processes, namely angiogenesis, adhesion and migration, and epithelial to mesenchymal transition (EMT), for which significantly regulated genes were identified. (b) Expression levels of the genes differentially expressed in MCF‐7, SK‐BR‐3, and MDA‐MB‐231 due to cultivation in FN‐silk network for 7 days. Values are represented as log2 fold‐change of the mean ± SD (n = 2, 3 technical replicates). Fold‐changes were calculated compared to the control 2D on day 1. For the statistical analysis, 2‐way ANOVA with Sidak correction was done. *P < 0.05; **P < 0.01; ** [file BTM2-8-e10537-s001.pdf]

Supplementary materials and methods

Cell culture

Three cell lines, MDA-MB-231, SKBR-3, and MCF-7, were obtained from American Type Culture Collection (ATCC) and cultured with a media mixture of DMEM low glucose (Thermo Fisher, 11574446), supplemented with 10% fetal bovine serum (Thermo Fisher, 16140- 071) and 1% penicillin-streptomycin 10.000 U/ml (Thermo Fisher, 11548876). The Wood and PB cell lines, kindly donated by Cellaria, were cultured in RETM basal medium (Cellaria, CM-0001) supplemented with 5% heat-inactivated fetal bovine serum (Hyclone, SH3007103HI), 3% RETM supplement, 1% penicillin-streptomycin 10.000 U/ml, and Cholera toxin with a final concentration of 0,025 µg/ml (EMD Millipore, 227036). Regular passaging of the cell cultures with TrypLE (Thermo Fisher, 12605-028) was performed when cells reached a confluency of around 80%. The medium was changed every other day, and all cells were kept in a cell incubator at 37°C and 5% CO^2. Cell counting was performed using a Bürker chamber (0,100 mm; 0,0025 mm^2; 10^4).

Patient-derived cells isolation

In 2021, material from 10 breast cancer patients was collected at Karolinska University Hospital. All procedures were approved by the local ethical committee and performed under the ethical permit 2016/957-31 (Main application approval), 2017/742-32 (Amendment). Biopsies were obtained via superficial scrapings from breast tumors as previously described by Ma et al.[1] and frozen in 10% DMSO solution. Cryovials were thawed at 37°C. Biopsy material was resuspended in warm DMEM/F-12, GlutaMAX medium (Thermo Fisher, 10565018) supplemented with 10% fetal bovine serum (FBS) and 1% penicillin-streptomycin 10.000 U/ml. The cell clumps were washed with a warm medium twice (370 g, 5 min). After the second wash, to enzymatically dissociate the cells, the cell pellet was incubated for 15 min in a cell incubator at 37°C and 5% CO^2 in a medium containing prediluted dispase 1U/ml (Stemcell, 07923). During the incubation, cell clumps were gently mixed every 3 minutes. Following the incubation, a medium containing FBS was added to block the enzymatic digestion. The cell suspension was then filtered through a 100 µm strainer. Cells were counted and seeded on 96-well plate TC treated (Thermo Fisher, 174929) or in FN-silk networks as described in the next section. The number of seeded cells was adapted for each biopsy, depending on the initial number of available cells. One sample (patient 3) was excluded because all cells were found dead after the dissociation. For two samples (patients 2 and 4), only a couple of cells were detected in the Burker chamber during cell counting, with an approximated starting material between 13.000 to 20.000 cells.

Alamar Blue proliferation assay

The metabolic rate of cells grown in tissue-culture treated 96-well plate or FN-silk network was measured using Alamar Blue cell viability reagent (Thermo Fisher, DAL1100) after 1, 3, and 7 days in culture. Briefly, after removal of the old culturing medium, 2D and 3D models were incubated for two hours at 37°C with 180 µl of Alamar Blue dye mixture diluted 1:10 in culturing medium. Three wells with only Alamar Blue were used as blank. After incubation, 100 µl of media were transferred to a new 96-well plate (Greiner, 655161), the plate was read using a plate reader (CLARIOstar, BMG Labtech) with excitation and emission wavelength of 544 nm and 595 nm, respectively. Fluorescence intensities of each well were obtained. For data analysis, the mean value of the blank was subtracted from each fluorescence intensity. For each condition, the average value and the standard deviation were calculated and plotted to compare 2D and FN-silk network cultures.

Live/Dead viability assay

Cells obtained from the superficial scraping of tumors and cultured in 2D or FN-silk networks for 7 days were stained using the LIVE/DEAD™ viability/cytotoxicity kit (Invitrogen, L3224). After medium removal, 200 µl of a warm medium containing 0,05% calcein and 0,2% ethidium homodimer-1 (EthD-1) was added to each well or scaffold. Cells were kept in the incubator for 25 min, and afterward, pictures were taken with the ANDOR camera, Leica DMI6000B. Images were captured using the NIS elements BR software.

Stereo microscope pictures for FN-silk network area quantification

A Nikon stereo microscope (SMZ 745T) was used to obtain macroscopic images of the FN-silk free-floating networks. The images were taken with a 1X magnification and analyzed with ImageJ to calculate the area of the FN-silk network model. For this analysis, the outer surface of the network was considered. The scale was set to 0,126 pixels/µm, based on the scale bar value obtained from the microscope.

Brightfield microscopy of FN-silk network

An inverted phase contrast microscope (Nikon, TMS-F) equipped with a camera at 4X was used to obtain macroscopic pictures of the FN-silk network changes over the seven days of culture.

Actin filaments staining of FN-silk network

Phalloidin staining was done to visualize actin filaments (F-actin) of cells grown in 2D and FN-silk network.

Samples were washed once with PBS and fixed with 4% paraformaldehyde (PFA) in PBS for 10 min at room temperature (RT) followed by three washes with PBS. The fixed cells were then permeabilized with 0,2% Triton X-100 in PBS for 15 min at RT. This was followed by two washes in wash buffer, Tween-20 (0,1%) in PBS, and by blocking with 1% bovine serum albumin (BSA) in PBS for 20 min at RT. Samples were then incubated for 40 min with phalloidin conjugated to Alexa Fluor 488 (Invitrogen, A12379) diluted 1:400 in PBS. An incubation step of 15 minutes with Sudan Black (Sigma Aldrich, 199664) 0,3% (w/v) working solutions dissolved in 70% ethanol was performed for FN-silk networks to quench the autofluorescence signal from the silk.[2] Both models were washed three times with wash buffer before nuclear staining with 4',6-Diamidine-2'-phenylindole dihydrochloride ((DAPI) Sigma Aldrich, 10236276001) diluted 1:1000 in PBS for 5 min. One final wash was done before imaging with the ANDOR camera, Leica DMI6000B. Images were captured using the NIS elements BR software.

Immunofluorescence staining

Cells fixed as described in the previous section were permeabilized with 0,2% Triton X-100 in PBS for 15 min at RT, followed by blocking with 10% goat serum in PBS (*i.e.* block buffer) for 1 h. An overnight incubation in a humidified chamber at 4°C was done using primary antibodies diluted in a mixture of 50% block buffer and 50% wash buffer (0,1% Tween-20 in PBS). The antibodies used were: HER2 (HPA, HPA001338, clone 181109, 117P) diluted 1:250 and ERα (Invitrogen, MA5-14501, clone SP1) diluted 1:150. The following day, three 5 min washes with washing buffer were performed, followed by 1 h incubation at RT shielded from light with Alexa 488-labeled anti-mouse secondary antibody (Invitrogen, A32723) and Alexa 546-labeled anti-rabbit (Invitrogen, A11010) diluted 1:500 in a mixture of 50% block buffer and 50% wash buffer. After the incubation with secondary antibodies, FN-silk networks were washed once in wash buffer before an incubation step with Sudan Black (Sigma Aldrich, 199664) for 15 min. Both models were washed three times with wash buffer, and nuclei were

stained with DAPI diluted 1:1000 in PBS for 5 min. Two final washes of 5 min in PBS were performed before imaging.

Formalin-fixed paraffin-embedded (FFPE) samples preparation

FN-silk network samples were kept in culture for seven days. On day 7, samples were washed once with PBS and fixed in 4% PFA for 15 min at RT. After fixation, three washes of 5 min each in PBS were performed. To enable an easier localization of the samples during sectioning, FN-silk networks were incubated in a 1:1 solution of PBS: Mayer's Hematoxilyn (Sigma, MHS32) for 30 s, followed by three 2 min washes with PBS. To maintain the integrity of the specimens, samples were pre-embedded in HistoGel (ThermoScientific, HG-4000-012). Briefly, a tube with solid HistoGel was heated to 60°C, and 150 µl of the liquid HistoGel was used to pre-coat a cryomold (10x10x5 mm). The FN-silk network was placed on top of the pre-coating and overlaid with an additional 200 µl of HistoGel. The cryomold was put on ice for 15 min for the Histogel to set. Pre-embedded samples were placed in a tissue embedding cassette and submerged in 70% ethanol. The samples were dehydrated using an automatized robot (Miles, Tissue-Tek V.I.P. E150/E300 Series). The program followed for dehydration consisted of two changes of 70% ethanol (30 min and 1 h), three changes of 95% ethanol (45 min, 45 min, and 1 h), and three changes of 99% ethanol (45 min and two of 1 h), two changes of xylene (1 h and 1 h 20 min), all performed at 40°C. These steps were followed by three changes of paraffin (two of 1 h and a third of 2 h) at 60°C. Finally, samples were embedded in paraffin using a tissue-tek embedding station (Microm, EC 350-1) and sectioned using a microtome (Microm, HM 360) to 12 µm thick sections.

Hematoxylin and eosin staining

FFPE sections were deparaffinized with two 5 min changes of xylene (Sigma, 534056) followed by 5 min in 100% ethanol, 2 min in 95%, and 2 min in 70% ethanol. Sections were briefly washed in distilled water and stained for 2 min in Harris-modified hematoxylin solution (Merck, HHS32). Sections were then rinsed under tap water for 1 min, differentiated with two dips in 1% HCl in 70% ethanol, rinsed with tap water, and blued in Scott´s tap water. Samples were then placed in 95% ethanol for 30 sec before counterstaining with 0,5% eosin for 3 min (Sigma, 861006), followed by rinsing in tap water. Dehydration was completed with sequential 1 min washes in 95% ethanol, 100% ethanol, and two changes of xylene. Sections were mounted with Permount mounting medium (VWR, 100496-552) and sealed with transparent nail polish.

RNA extraction and cDNA synthesis

The medium was removed, and cells were washed once with PBS and lysed through the addition of cell lysis buffer for 2D (RNeasy minikit, QIAGEN, 74104) or FN-silk networks (RNeasy microkit, QIAGEN, 74034) to obtain a volume of 350 µl total. 2D cultures were collected by scraping the bottom of the 6-well plate with a cell scraper, the cells were collected in an Eppendorf tube. For the 3D cultures, five or three FN-silk networks were pulled together for lysis on day 1 and day 7, respectively. A syringe with a needle diameter of 0,4 µm was used to homogenize the samples via mixing 5-6 times, lysates were then transferred to an Eppendorf tube. Total RNA was extracted from lysed 2D models by using RNeasy minikit. For the FN-silk network, the silk debris was removed via centrifugation (12.000 g, 5 min), the supernatant was then processed using the RNeasy microkit. To measure the purity and quantity of RNA obtained, Nanodrop measurements were performed. Synthesis of cDNA was done using PrimeScript RT Kit (TaKaRa, RR037A) accordingly to the manufacturer's instructions.

RT-qPCR

Reverse transcription quantitative real-time PCR (RT-qPCR) reactions were performed in a CFX96TM Real-Time System, C1000TM Thermal Cycler from BIO-RAD using SYBR Green Universal Assay (BIO-RAD, 1725271). In each RT-qPCR well, 18 µL of mix were added, the mix contained 10 µl of SYBR Green 2X, 0,6 µl of a forward and reverse primer at 10 µM, and 7,4 µl of water. The primers had a final concentration of 0,3 µM/reaction. 2 µl containing 10 ng of cDNA were added to each well. Normalized values were calculated by dividing the mean expression value by a factor equal to the geometric mean of the normalization genes (*i.e.* beta-2 microglobulin (*B2M*), transferrin receptor (*TFRC*) for MCF-7, SK-BR-3, MDA-MB-231, *B2M* and glyceraldehyde-3-phosphate dehydrogenase (*GAPDH*) for the Wood cells, and *B2M* for PB cells) and applying the ΔΔCt method.[3] For a precise calculation, normalized values were obtained by dividing the expression value by a factor equal to the geometric mean of the normalization genes. Table 1 contains the complete list of primers used in this study.

RNA-sequencing and data processing

For each condition, to ensure reliable statistical power, three independent replicates were prepared. To guarantee the extraction of enough total RNA from the samples lysed on day seven, five FN-silk networks were pulled together for each replicate. RNA integrity was determined by capillary electrophoresis on an Agilent 2100 Bioanalyzer using the Eukaryote Total RNA Nano assay, an RNA Integrity Number (RIN) major than 6 was observed for all samples. 200 ng of total RNA was used to generate stranded mRNA libraries with the mRNA prep ligation kit according to the manufacturer's instructions (20040534, Illumina, USA). The concentration and quality of the cDNA libraries were assessed by Qubit (Life Technologies, USA) and TapeStation, respectively. Sequencing was performed with Illumina RNA-seq (Nextseq 2000, P2 flowcell, Paired End, dual index). For data processing, Illumina bcl2fastq (v2.20.0) was used for base calling and demultiplexing. Read quality was checked for each sample using FastQC (v0.11.8). Reads were aligned to Ensemble GRCh38/hg38 reference genome using STAR (v2.6.1d). Read summarization to gene counts was performed using featureCounts (v1.5.1). Normalized counts were generated using DESeq2 (v1.28.1). The principal component analysis (PCA) quality test results did not reveal any outliers. To identify the genes modulated by cultivation on FN-silk, we performed, for each cell line, two pairwise differential analyses comparing a) cells cultured in FN-silk network or 2D for seven days (*i.e.*, FN-silk network *vs* 2D at day 7) and b) cells cultured in FN-silk network for seven days as opposed to the initial cell population harvested from the tissue-culture treated flask (*i.e.*, FN-silk network day 7 *vs* control flask at day 0). For both comparisons, we applied a conservative significance threshold of 5% to the corrected *p*-value, associated with a log2 fold-change $\geq |\pm 0{,}38|$. A gene was considered as differentially expressed (DEG) by culture in 3D when significantly modulated in both comparisons.

Statistical analysis

For the statistical analysis, 2-way ANOVA with interaction was used to analyze the data in GraphPad. Differences between groups were considered statistically significant when P-value < 0,05.

References

1. Ma R, Fredriksson I, Karthik GM, et al. Superficial scrapings from breast tumors is a source for biobanking and research purposes. *Lab Invest.* 2014;94(7):796-805.
2. Neo PY, Tan DJ, Shi P, Toh SL, Goh JC. Enhancing analysis of cells and proteins by fluorescence imaging on silk-based biomaterials: modulating the autofluorescence of silk. *Tissue Eng Part C Methods.* 2015;21(2):218-228.

3. Kenneth J. Livak TDS. Analysis of Relative Gene Expression Data Using Real-Time Quantitative PCR and the $2^{-\Delta\Delta CT}$ Method. *ELSEVIER*. 2001;25(4):402-408.

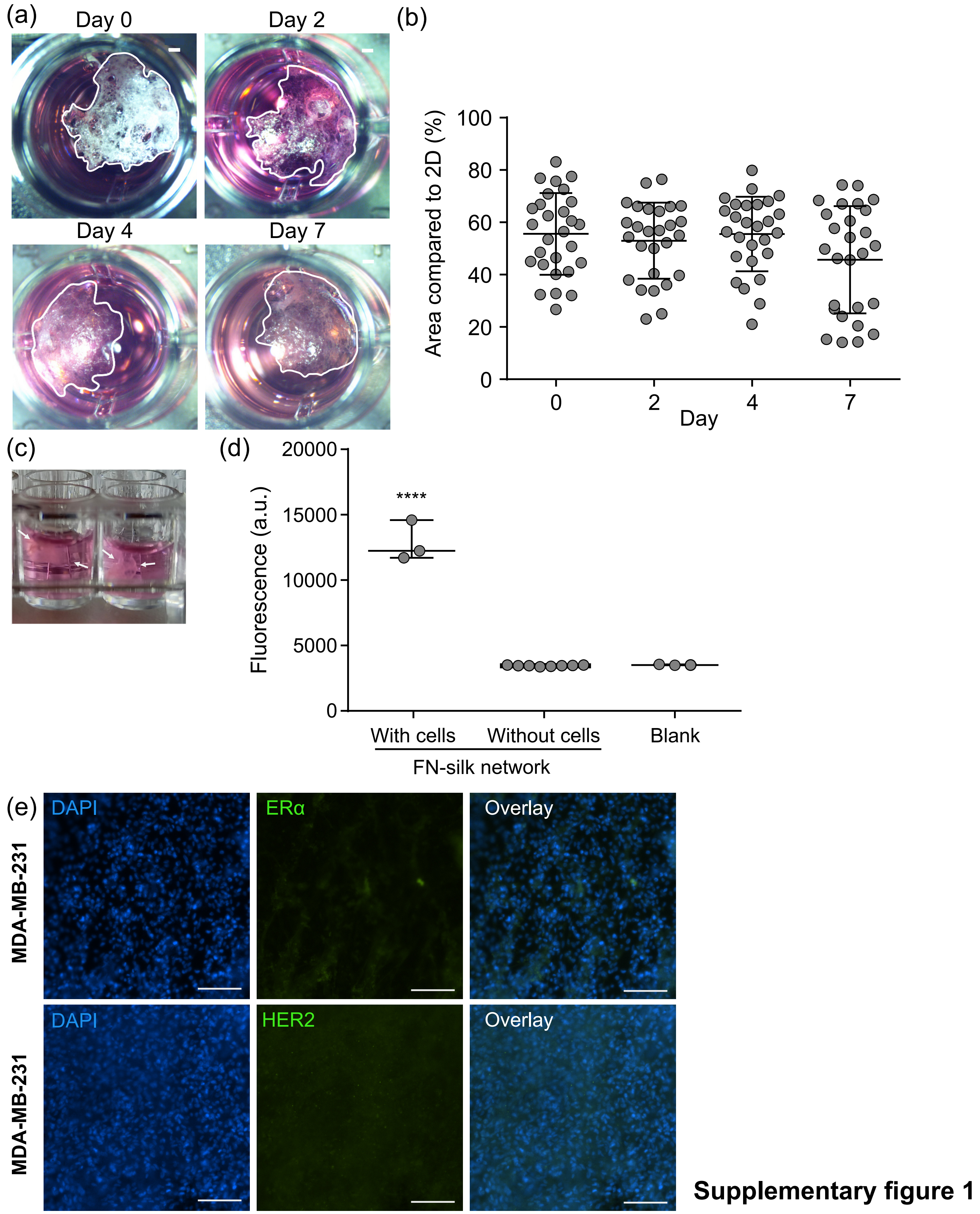

Supplementary fig. 1. Additional characterization of FN-silk network constructs and negative control staining for ERα and HER2. (a) Representative stereo microscope pictures of the full FN-silk networks were taken on days 0, 2, 4, and 7. The white line indicates the border of the FN-silk network, which was drawn to determine the area of each scaffold (n=3, with 9 technical replicates). Scale bar: 500 µm. (b) Grouped plots display the size of the FN-silk network areas as a percentage compared to the surface of a 96-well plate well. Mean ± SD, with individual data points shown (n=3 with 9 technical replicates per independent experiment). (c) Pictures of two wells from a 96-wp in which FN-silk networks (day 2) are floating in the mid-height part of the well. The white arrows point to the floaters. (d) Graph showing the fluorescence detected after incubation with 10% Alamar Blue medium in FN-silk network with cells (3 technical replicates) as compared with control without cells (8 replicates) and empty wells (3 replicates). (e) Immunofluorescence staining for ER, (green), HER2 (green), and nuclei (DAPI, blue) in MDA-MB-231 cells cultured in the FN-silk network for 7 days (n=2, 3 technical replicates). Single channels and overlay images are shown. Scale bar: 100 µm.

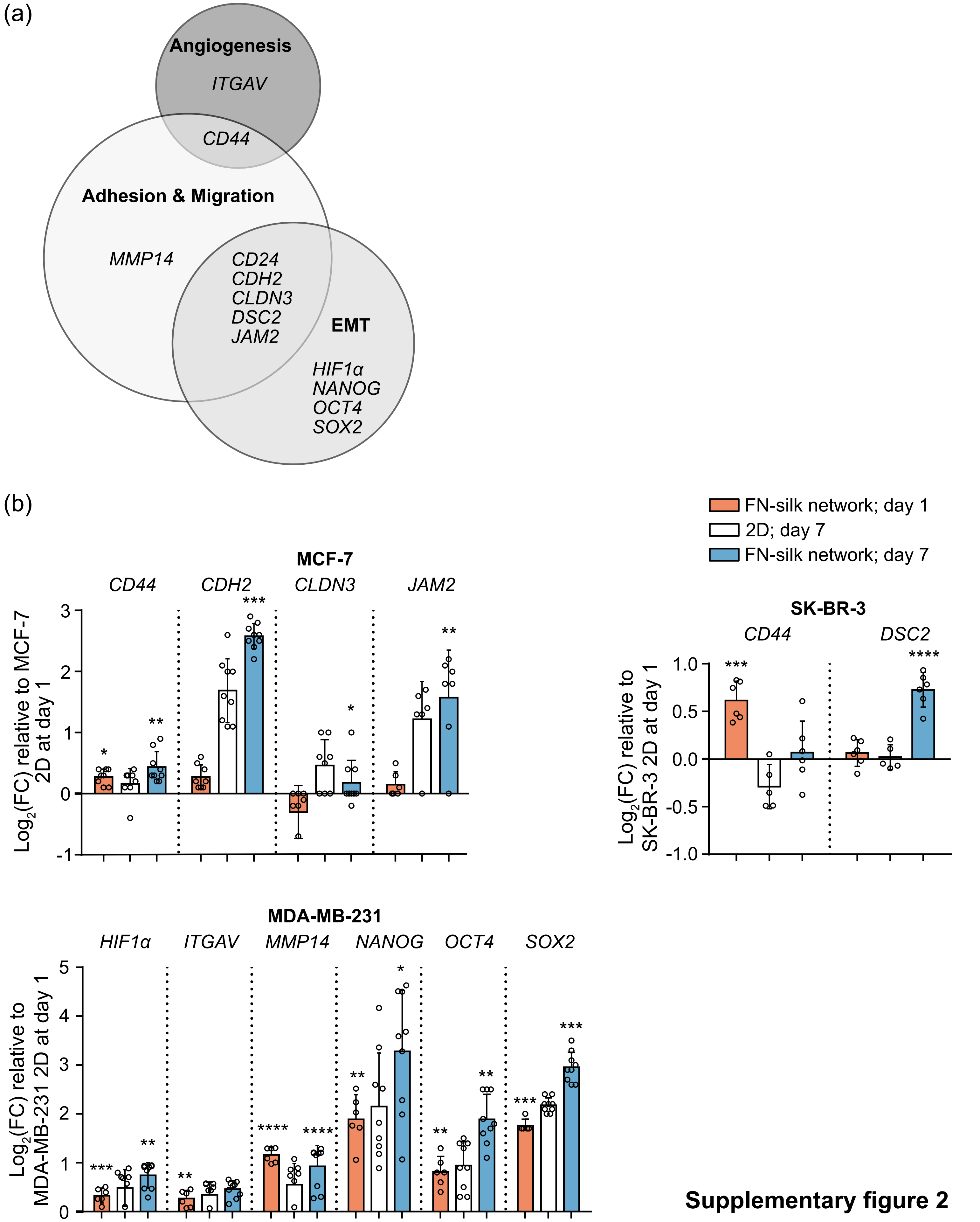

Supplementary fig. 2. RT-qPCR analysis of gene expression changes driven by FN-silk network. (a) Venn diagram representing the three biological processes, namely angiogenesis, adhesion and migration, and epithelial to mesenchymal transition (EMT), for which significantly regulated genes were identified. (b) Expression levels of the genes differentially expressed in MCF-7, SK-BR-3, and MDA-MB-231 due to cultivation in FN-silk network for seven days. Values are represented as log2 fold-change of the mean ± SD (n=2, 3 technical replicates). Fold-changes were calculated compared to the control 2D on day 1. For the statistical analysis, 2-way ANOVA with Sidak correction was done. *$P < 0{,}05$; **$P < 0{,}01$; ***$P < 0{,}001$; ****$P < 0{,}0001$.

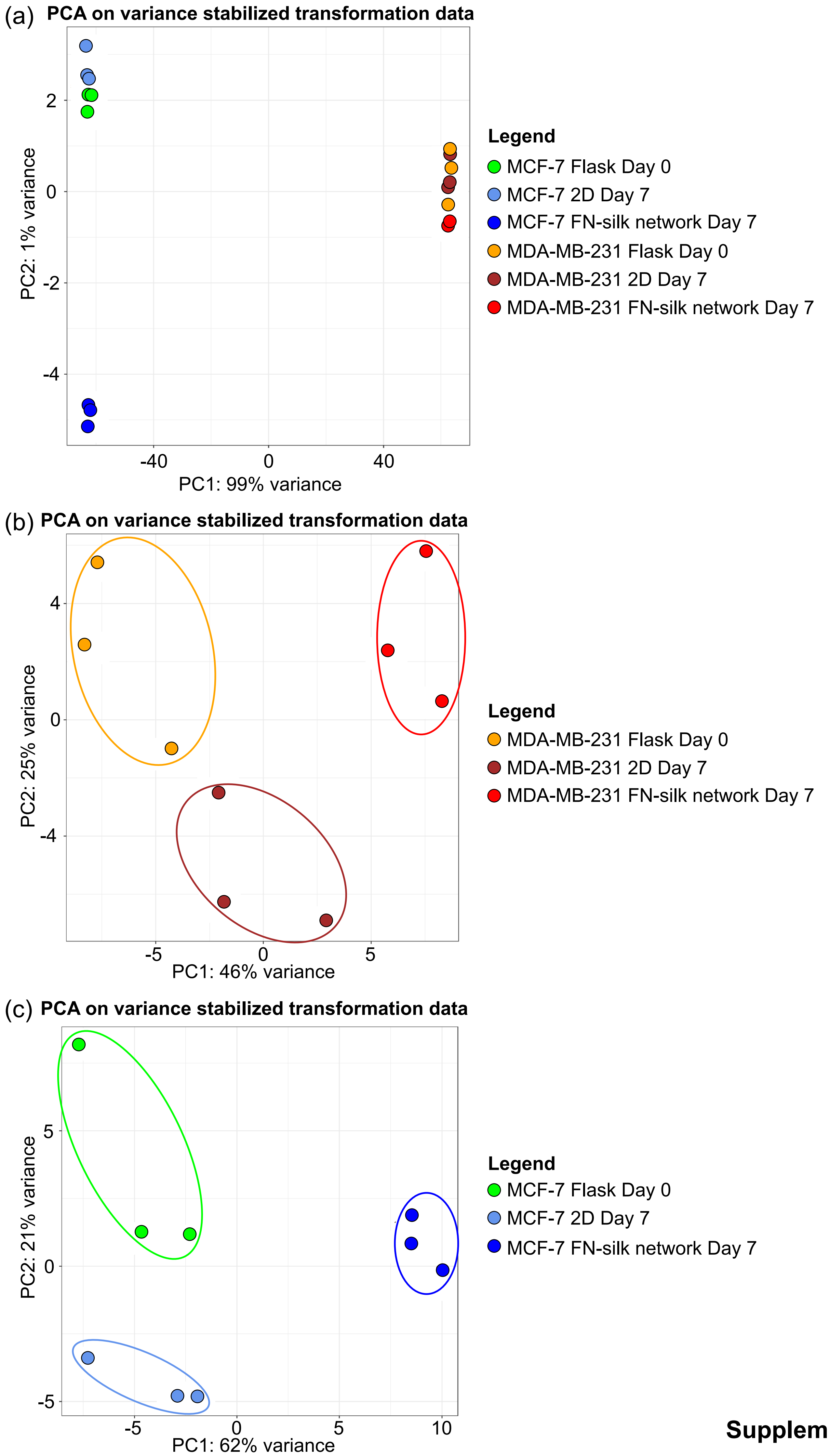

Supplementary figure 3

Supplementary fig. 3. Principal component analysis (PCA) score plots. (a) PCA score plot based on the global gene expression data generated from comparing MCF-7 and MDA-MB-231 harvested at day 0 from the flask or at day 7 after culture in 2D or FN-silk network. The first two principal components (PC1 and PC2) explain 99% and 1% of the total variance in the data and correspond to cell type and FN-silk network effect on MCF-7, respectively. (b) PCA score plot was obtained from the gene expression dataset solely of MDA-MB-231 samples. The FN-silk network after seven days in culture is responsible for 46% of the total variance in the data, as shown by PC1. (c) PCA score plot representing the MCF-7 subset of samples. The FN-silk network effect after seven days in culture accounts for 62% of the total variance in the data. Samples are depicted as indicated in the figure. N=3 for each cell line.

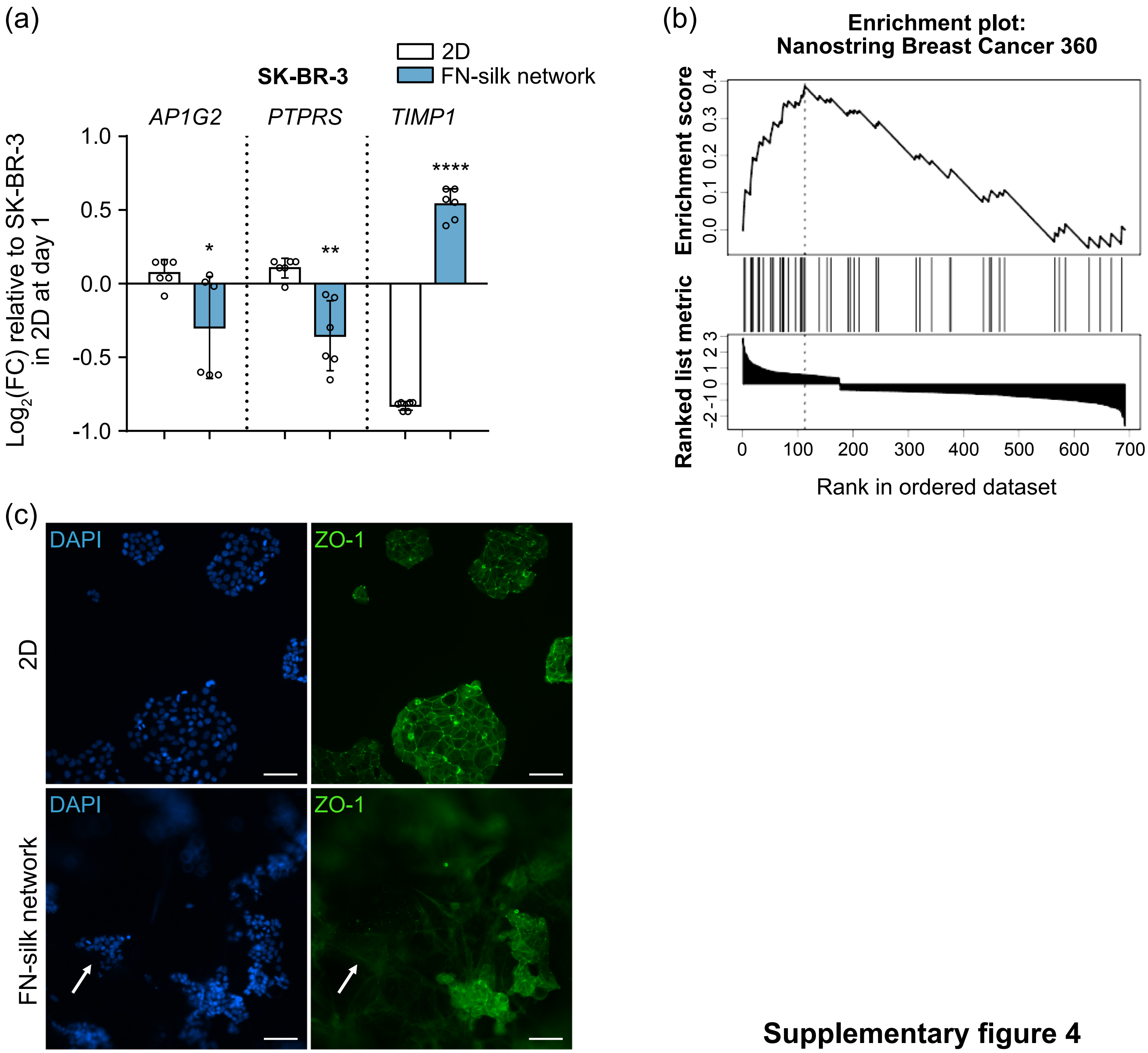

Supplementary figure 4

Supplementary fig. 4. FN-silk network targets, further investigation at transcriptional and protein levels. (a) mRNA levels of genes commonly regulated by growth on FN-silk network were compared in SK-BR-3 grown in 2D or FN-silk network for 7 days *vs* at day 1 in 2D. Values are represented as log2 fold-change of the mean ± SD (n=2, three technical replicates). For the statistical analysis a t-test was done. *$P < 0,05$; **$P < 0,01$; ****$P < 0,0001$. (b) Gene set enrichment analysis (GSEA)-enrichment plot revealing a significant enrichment score when comparing the FN-silk network signature in MCF-7 with the gene panel of Nanostring Breast Cancer 360. (c) Staining of ZO-1 and nuclei done on MCF-7 cultured in 2D or FN-silk network for 7 days. Single channels are shown. n=2, with three technical replicates. Scale bar: 100 µm.

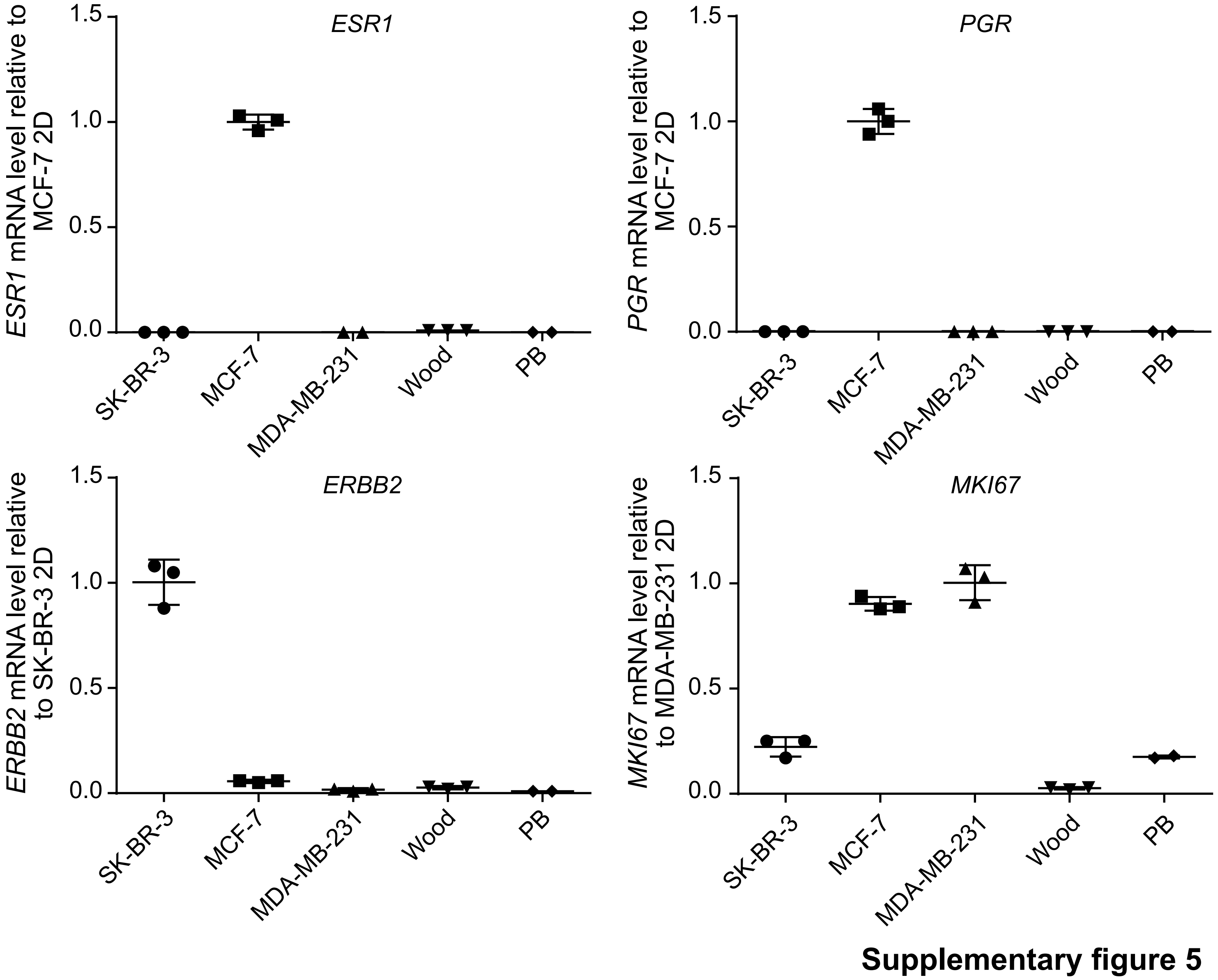

Supplementary fig. 5. Breast cancer markers in Wood and PB cells. Gene expression levels of the canonical breast cancer markers *ESR1, PGR, ERBB2,* and *MKI67* were measured in SK-BR-3, MCF-7, MDA-MB-231, Wood, and PB cells kept in culture in 2D. Values are represented as fold-change of the mean ± SD (n=3). Fold-change was calculated comparing MCF-7 for *ESR1* and *PGR*, SK-BR-3 for *ERBB2*, and MDA-MB-231 for *MKI67*.
